# Supplementary material for: Tuned SMC Arms Drive Chromosomal Loading of Prokaryotic Condensin
Source: Mol Cell. 2017 Mar 2;65(5):861–872.e9. doi: 10.1016/j.molcel.2017.01.026 (PMC5344682; doi:10.1016/j.molcel.2017.01.026)
Supplement: Document S1. Figures S1–S6 and Tables S1–S3 [file mmc1.pdf]

Molecular Cell, Volume *65*

Supplemental Information

Tuned SMC Arms Drive Chromosomal Loading of Prokaryotic Condensin

Frank Bürmann, Alrun Basfeld, Roberto Vazquez Nunez, Marie-Laure Diebold-Durand, Larissa Wilhelm, and Stephan Gruber

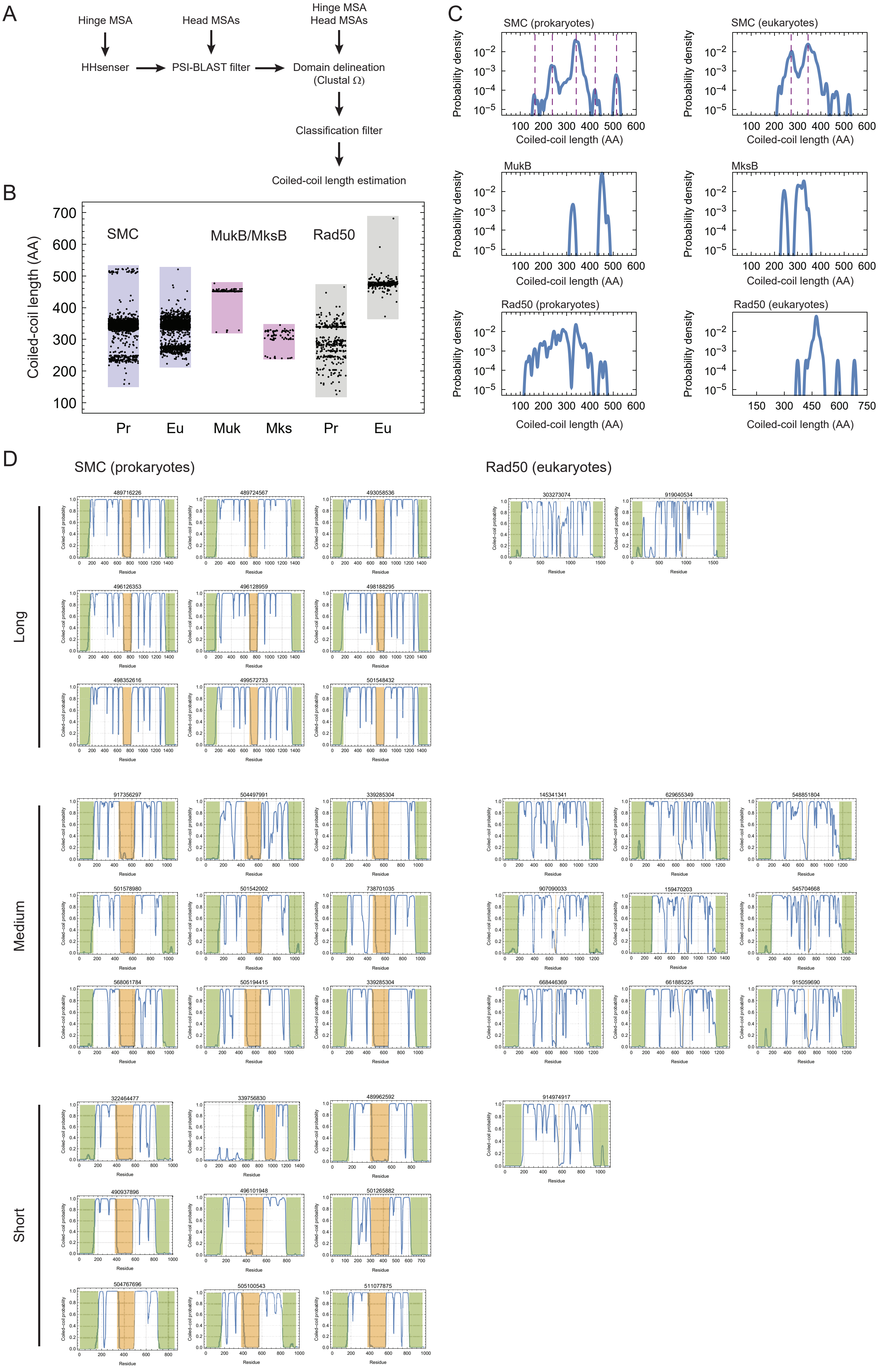
A
Hinge MSA
Head MSAs
Hinge MSA
Head MSAs
HHsenser
PSI-BLAST filter
Domain delineation
(Clustal Ω)
Classification filter
Coiled-coil length estimation
B
Coiled-coil length (AA)
SMC
MukB/MksB
Rad50
Pr
Eu
Muk
Mks
Pr
Eu
C
SMC (prokaryotes)
SMC (eukaryotes)
MukB
MksB
Rad50 (prokaryotes)
Rad50 (eukaryotes)
Probability density
Coiled-coil length (AA)
D
SMC (prokaryotes)
Rad50 (eukaryotes)
Long
Medium
Short
Coiled-coil probability
Residue

Figure S1

Figure S1. Coiled-coil length distributions of SMC and SMC-like proteins. Related to Figure 1.

(A) Flow scheme for the estimation of coiled-coil length distributions.

(B) Arm length distributions for different groups of SMC and SMC-like proteins. Also shown in Figure 1B: SMC prokaryotes, n = 3337; SMC eukaryotes, n = 1659. Additional data: MukB, n = 179; MksB, n = 75; Rad50 prokaryotes, n = 365; Rad50 eukaryotes, n = 306.

(C) Kernel density estimates for data shown in B. Top panels also shown in Figure 1C.

(D) Example profiles for selected SMC family sequences with short, medium and long coiled-coil arms. Marcoil coiled-coil predictions are shown in blue colors, and regions identified by the sequence alignment approach described in A are indicated in green (HeadN and HeadC) and orange (Hinge), respectively. GenBank identifiers are indicated on top of the plots.

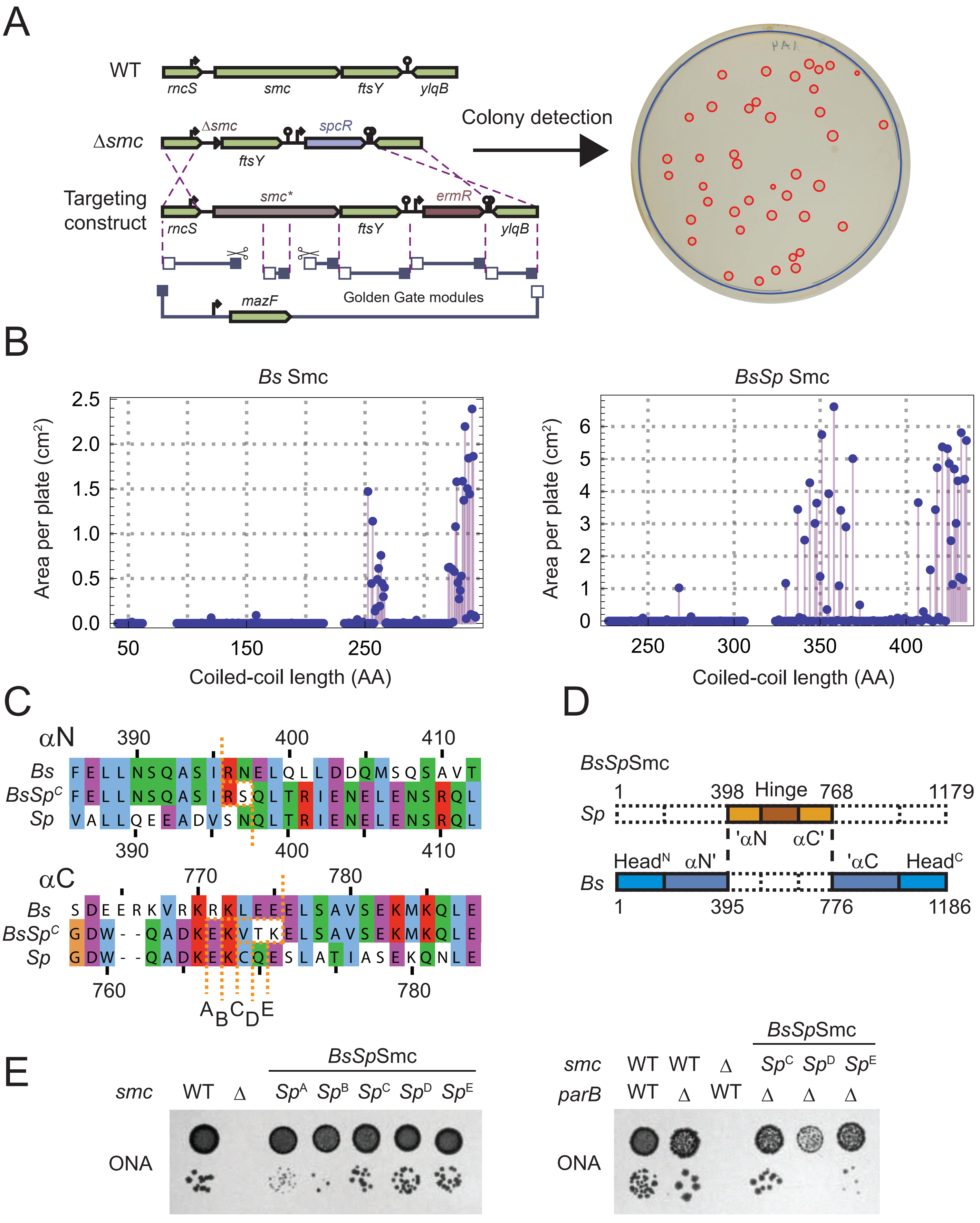

A
WT
rncS
smc
ftsY
ylqB
Δsmc
Δsmc
spcR
ftsY
Colony detection
Targeting construct
smc*
ermR
rncS
ftsY
ylqB
mazF
Golden Gate modules
B
Bs Smc
BsSp Smc
Area per plate (cm²)
Coiled-coil length (AA)
C
αN
αC
Bs
BsSp[C]
Sp
A B C D E
D
BsSpSmc
Sp
Bs
Hinge
'αN
αC'
Head[N]
αN'
'αC
Head[C]
1
398
768
1179
1
395
776
1186
E
smc
WT
Δ
BsSpSmc
Sp[A] Sp[B] Sp[C] Sp[D] Sp[E]
ONA
parB

Figure S2

Figure S2. Coiled-coil truncation and extension screens. Related to Figure 2.

(A) Gene targeting strategy.

(B) Bacterial growth per plate obtained for the coiled-coil shortening screen (left panel) and the coiled coil extension screen (right panel) shown in Figure 2C.

(C) Sequence alignment for the seam of different *BsSp* chimeras. *Bs* Smc and *Sp* Smc were combined with slightly altered connections in the C-terminal coiled-coil strand (bottom), whereas the N-terminal strand was kept constant (top). Connections of the variants are indicated by dotted lines.

(D) Scheme for construction of the *BsSp* Smc hybrid protein used in Figure 2C.

(E) Spot dilutions for *BsSp* Smc variants in the presence (left) or absence (right) of the *parB* gene. Deletion of *parB* sensitizes strains with hypomorphic *smc* alleles (Gruber and Errington, 2009). Variant C was used for the screen shown in Figure 2C.

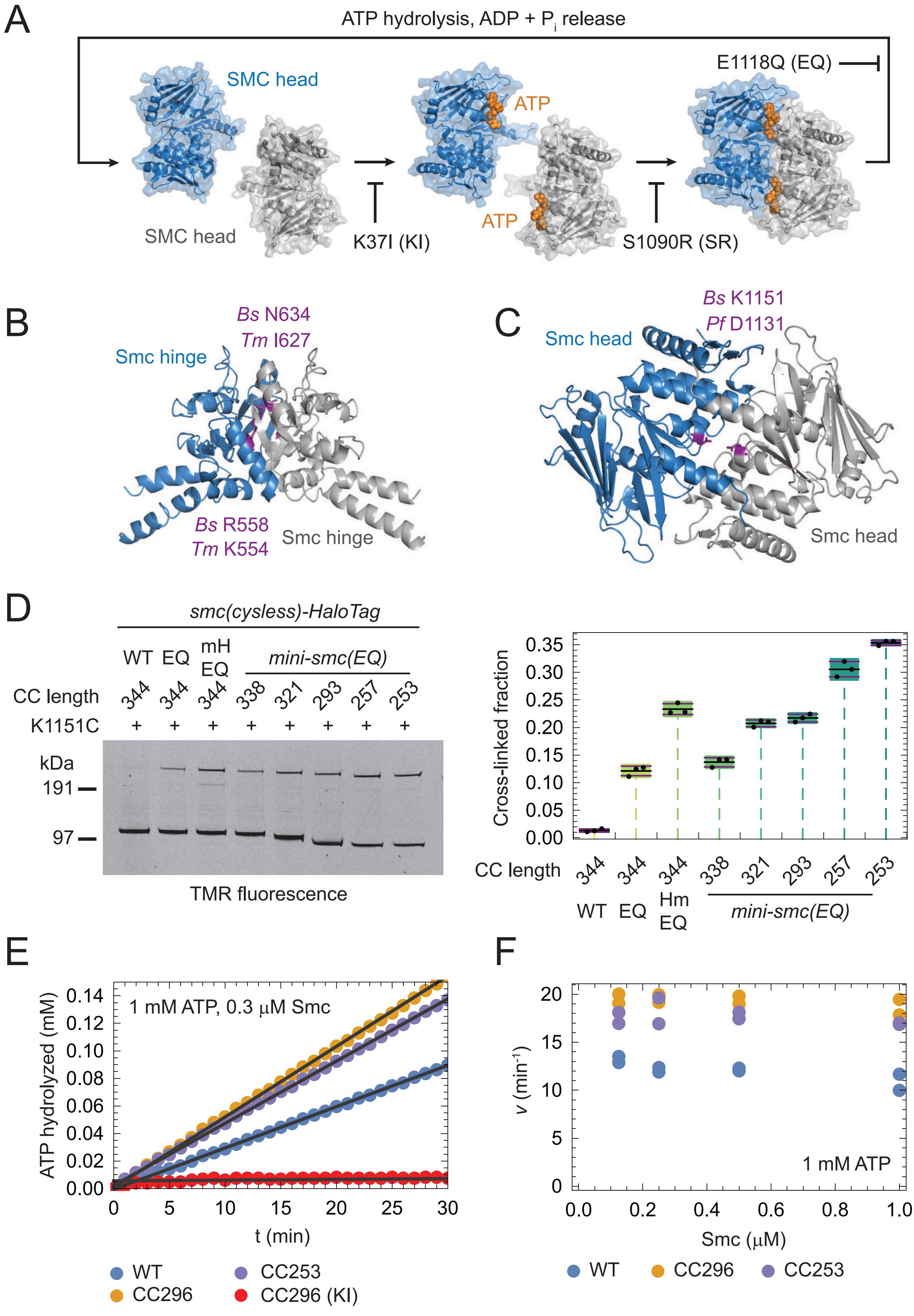
A
ATP hydrolysis, ADP + Pi release
SMC head
ATP
E1118Q (EQ)
SMC head
K37I (KI)
ATP
S1090R (SR)
B
Bs N634
Tm I627
Smc hinge
Bs R558
Tm K554
Smc hinge
C
Bs K1151
Pf D1131
Smc head
Smc head
D
smc(cysless)-HaloTag
WT EQ mH EQ
mini-smc(EQ)
CC length 344 344 344 338 321 293 257 253
K1151C + + + + + + + +
kDa
191
97
TMR fluorescence
Cross-linked fraction
0.35
0.30
0.25
0.20
0.15
0.10
0.05
0.00
CC length 344 344 344 338 321 293 257 253
WT EQ Hm EQ
mini-smc(EQ)
E
1 mM ATP, 0.3 μM Smc
ATP hydrolyzed (mM)
0.14
0.12
0.10
0.08
0.06
0.04
0.02
0.00
0 5 10 15 20 25 30
t (min)
WT
CC296
CC253
CC296 (KI)
F
v (min⁻¹)
20
15
10
5
0
1 mM ATP
0.0 0.2 0.4 0.6 0.8 1.0
Smc (μM)
WT
CC296
CC253

Figure S3

Figure S3. Site-specific cross-linking of interfaces in Mini-Smc variants. Related to Figure 3.

(A) Overview of the SMC ATPase cycle. *Bs* Smc mutations interfering with the indicated steps are: K37I (KI), blocks ATP binding; S1090R (SR), blocks head engagement; E1118Q (EQ), blocks ATP hydrolysis. Head structures are from (Lammens et al., 2004).

(B) The location of the reporter residues R558C/N634C (Bürmann et al., 2013) is mapped onto the *Tm* hinge crystal structure (PDB: 1GXL, top).

(C) The head engagement reporter residue K1151C (Minnen et al., 2016) is mapped onto the *P. furiosus* (*Pf*) head crystal structure (PDB: 1XEQ).

(D) *In vivo* site-specific cross-linking of Mini-Smc variants at the head interface. EQ, E1118Q allows ATP binding but impairs hydrolysis; mH, monomeric hinge (Hirano and Hirano, 2002; Minnen et al., 2016). As in Figure 3B.

(E) Examples of ATPase time course measurements. Representative data for selected Smc variants at 1 mM ATP are shown.

(F) Specific activity of selected Smc variants at 1 mM ATP and variable protein concentration.

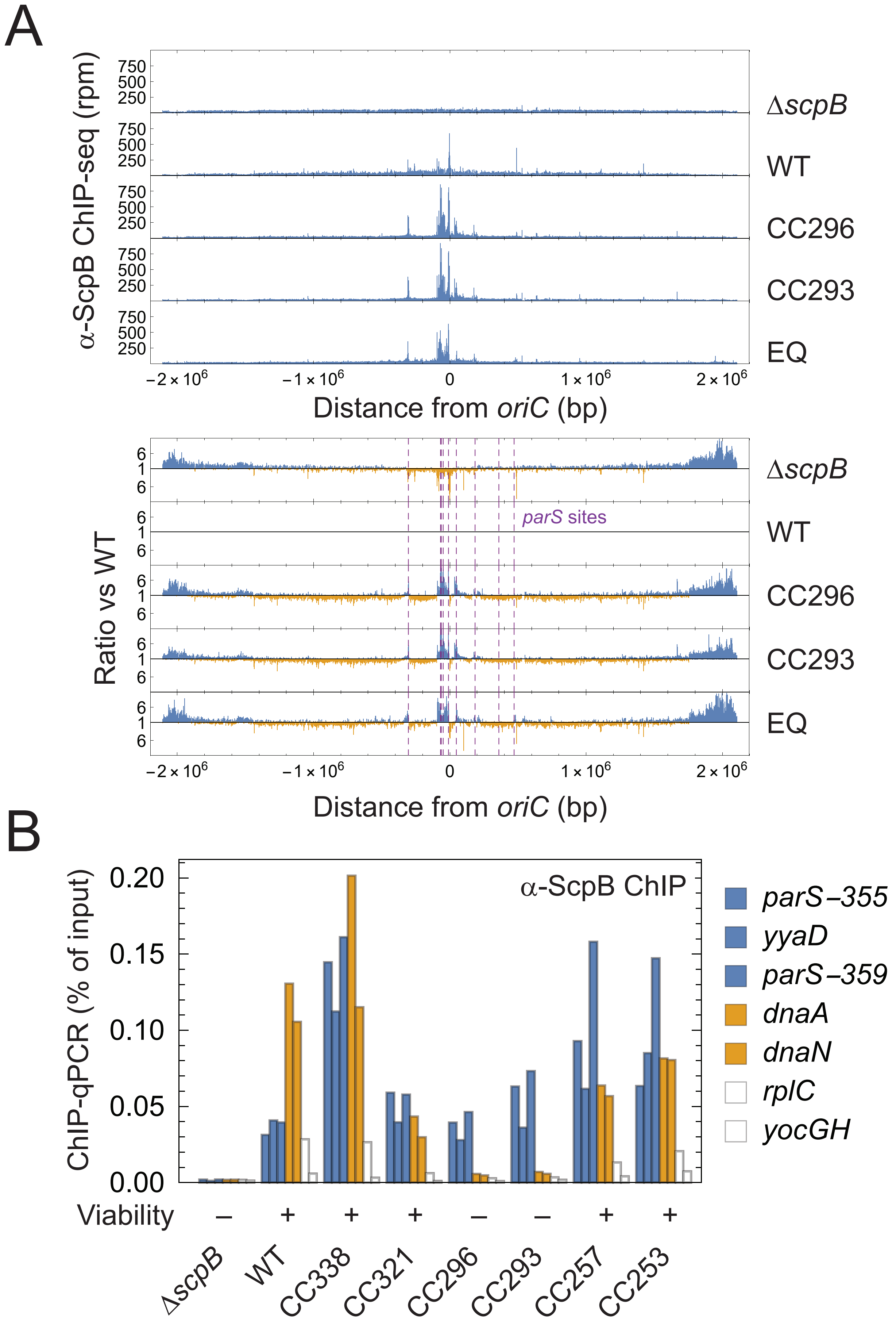
A
α-ScpB ChIP-seq (rpm)
750
500
250
ΔscpB
WT
CC296
CC293
EQ
−2 × 10^6
−1 × 10^6
0
1 × 10^6
2 × 10^6
Distance from oriC (bp)
Ratio vs WT
6
1
6
parS sites
B
ChIP-qPCR (% of input)
0.20
0.15
0.10
0.05
0.00
α-ScpB ChIP
parS−355
yyaD
parS−359
dnaA
dnaN
rplC
yocGH
Viability
–
+
ΔscpB
WT
CC338
CC321
CC296
CC293
CC257
CC253

Figure S4

Figure S4. Chromosomal localization of Mini-Smc variants. Related to Figure 4.

(A) Chromosome-wide ChIP-seq profiles for different Smc variants. Sequence distributions for the immunoprecipitate (top) and ratiometric analysis against the wild-type profile (bottom) are shown. Locations of *parS* sites are indicated by purple dashed lines.

(B) ChIP-qPCR against ScpB for *mini-smc* strains. Loci close to Smc loading sites are colored in orange, loci close to the replication origin are blue and chromosomal arm positions are white (see also Figure 4B).

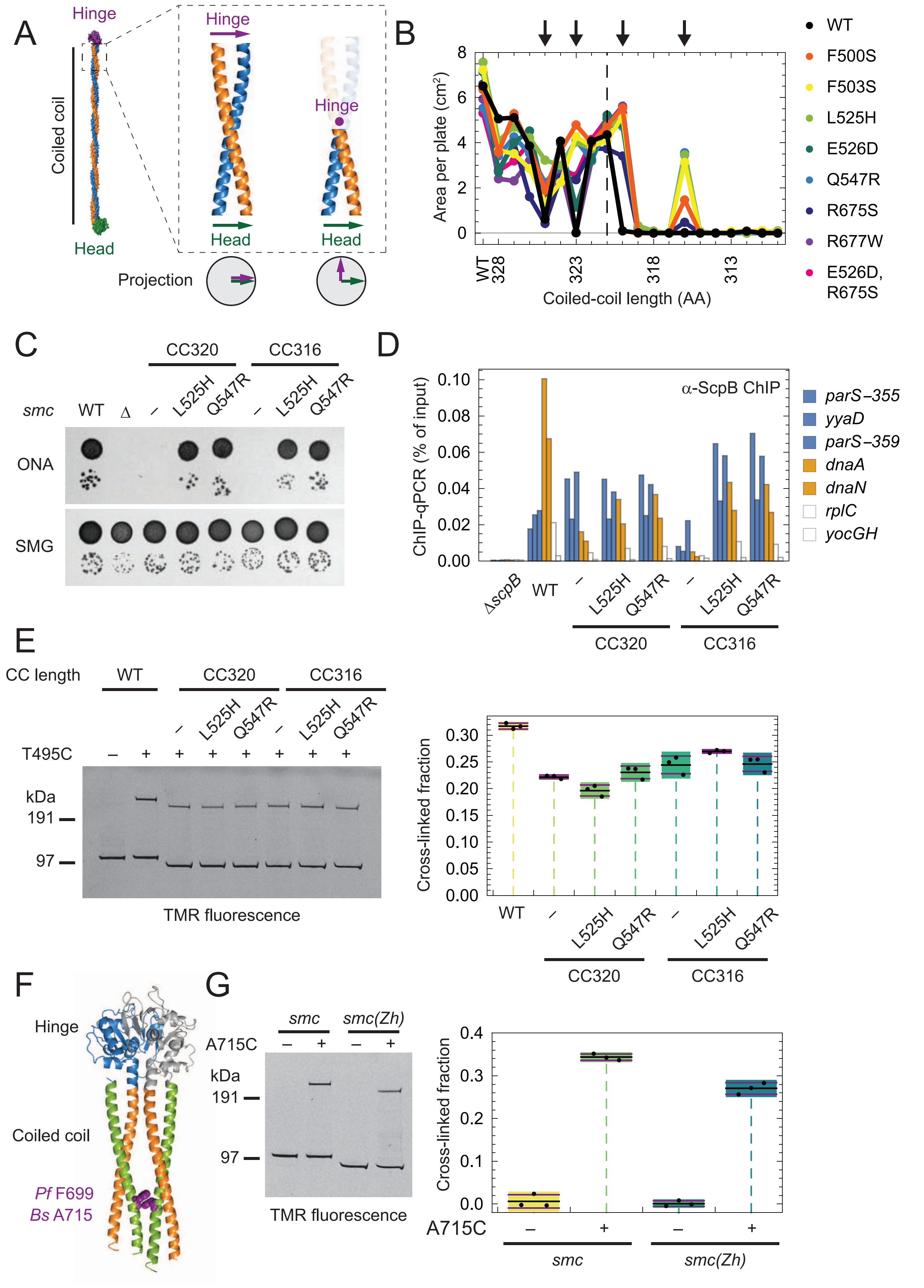
A
Hinge
Coiled coil
Head
Hinge
Head
Hinge
Head
Projection
B
Area per plate (cm²)
Coiled-coil length (AA)
WT 328
323
318
313
WT
F500S
F503S
L525H
E526D
Q547R
R675S
R677W
E526D, R675S
C
smc
WT
Δ
CC320
CC316
L525H
Q547R
ONA
SMG
D
ChIP-qPCR (% of input)
α-ScpB ChIP
parS-355
yyaD
parS-359
dnaA
dnaN
rplC
yocGH
ΔscpB
E
CC length
T495C
kDa
191
97
TMR fluorescence
Cross-linked fraction
F
Hinge
Coiled coil
Pf F699
Bs A715
G
smc
smc(Zh)
A715C
kDa
191
97
TMR fluorescence
Cross-linked fraction

Figure S5

Figure S5. Characterization of Mini-Smc suppressors and the Smc(Zh) chimera. Related to Figure 5.

(A) Modifications in Smc arm length might alter hinge/head orientation by changing the phase-relationship between coiled-coil ends.

(B) Confirmation of suppressor mutations. The indicated mutations were introduced into different *mini-smc* variants by an approach similar to the one shown in Figure 2A, and total colony area per plate of transformants was assessed.

(C) Spot dilutions of selected *mini-smc* suppressor strains. As in Figure 2E.

(D) ChIP-qPCR against ScpB for *mini-smc* strains containing suppressor mutations. Loci close to Smc loading sites are colored in orange, loci close to the replication origin are blue and chromosomal arm positions are white (see also Figure 4B).

(E) Site-specific *in vivo* cross-linking at the hinge-proximal coiled-coil residue T495C in suppressed Mini-Smc variants (Soh et al., 2015). As in Figure 3A.

(F) Rod conformation of the hinge-proximal coiled coil (*Pf* Smc, PDB: 4RSJ). The reporter residue A715C is indicated in purple (Soh et al., 2015). The region that has been modified in the truncation screen is shown in orange and green, the constant hinge region is shown in blue and gray. For experiments involving the Rad50 Zinc hook, the constant hinge region was replaced by the hook.

(G) Association of the hinge-proximal coiled coil in the Smc(Zh) chimera as determined by site-specific *in vivo* cross-linking. See Figure S5F for location of the reporter residue. As in Figure 3A.

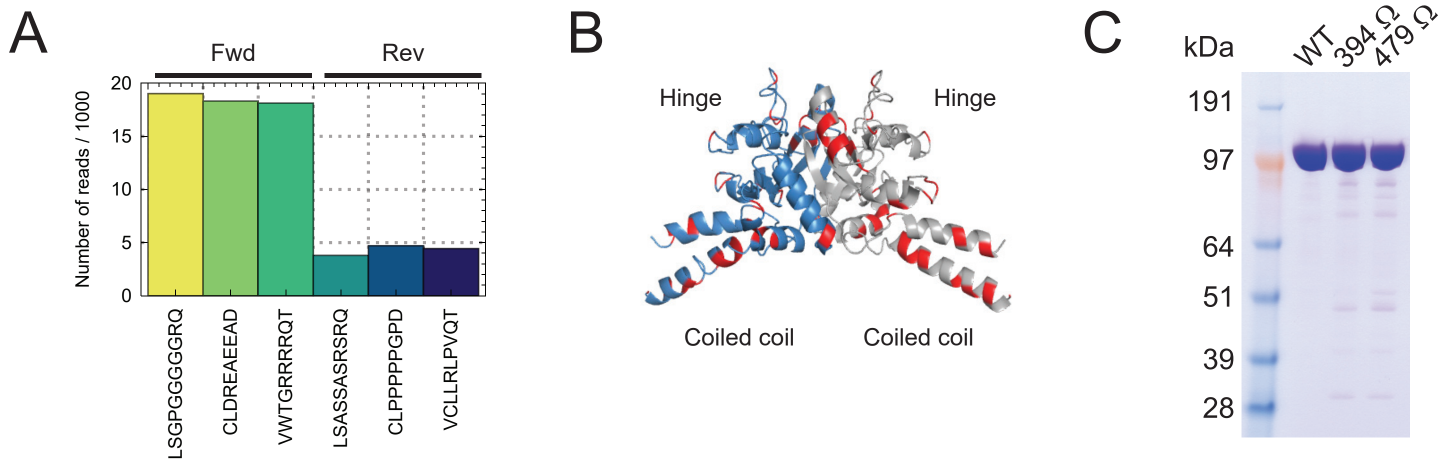
A
Fwd
Rev
Number of reads / 1000
20
15
10
5
0
LSGPGGGGRQ
CLDREAEEAD
VWTGRRRQT
LSASSASRSRQ
CLPPPPPGPD
VCLLRLPVQT
B
Hinge
Hinge
Coiled coil
Coiled coil
C
kDa
WT
394 Ω
479 Ω
191
97
64
51
39
28

Figure S6

Figure S6. Transposon insertion screen. Related to Figure 6.

(A) Read counts for all six different frames of the inserted sequence.
(B) Positions of peptide insertions resulting in functional proteins are mapped onto the *Tm* hinge structure (PDB: 1GXL).
(C) Purification of Smc variants with peptide insertions in the coiled-coil arm. Purified fractions were analysed by SDS-PAGE and Coomassie staining.

Supplemental tables

Table S1, related to Figures 1- 6. Strain usage.

Figure	Strains
2E	BSG1002, BSG1007, BSG2088, BSG2089, BSG2090, BSG2091, BSG2092, BSG2093, BSG2094, BSG2104
2F	BSG1002, BSG1007, BSG2348, BSG2349, BSG2355, BSG2350, BSG2351, BSG2352, BSG2353, BSG2356, BSG2354
3A	BSG1360, BSG1638, BSG2118, BSG2119, BSG2403, BSG2120, BSG2121, BSG2122
3B	BSG1457, BSG1488, BSG1600, BSG2511, BSG2531, BSG2408, BSG2135
4A	BSG1891, BSG1002, BSG2090, BSG2091, BSG1008
4B	BSG1891, BSG1002, BSG1008, BSG1046, BSG2090, BSG2409, BSG2091, BSG2410
4C	BSG1782, BSG1784, BSG1786, BSG2617, BSG2618, BSG2619, BSG2620
5B	BSG1002, BSG2479, BSG2578, BSG2579, BSG2580
5E	BSG1002, BSG1007, BSG1075, BSG2414, BSG2415, BSG2416, BSG2417, BSG2418, BSG2419
6B	BSG1002, BSG1007, BSG2017, BSG2018, BSG2021, BSG2026, BSG1835
6C	BSG1891, BSG1002, BSG2017, BSG2018, BSG2021, BSG2026, BSG1835
S3D	BSG1457, BSG1488, BSG1598, BSG2133, BSG2134, BSG2135, BSG2136, BSG2137
S4A	BSG1891, BSG1002, BSG2090, BSG2091, BSG1008
S4B	BSG1891, BSG1002, BSG2088, BSG2089, BSG2090, BSG2091, BSG2092, BSG2093
S5C	BSG1002, BSG1007, BSG2479, BSG2480, BSG2481, BSG2482, BSG2483, BSG2484
S5D	BSG1891, BSG1002, BSG2479, BSG2480, BSG2481, BSG2482, BSG2483, BSG2484
S5E	BSG1457, BSG2485, BSG2486, BSG2487, BSG2488, BSG2492, BSG2493, BSG2494
S5G	BSG1360, BSG1921, BSG2512, BSG2513

Table S2, related to STAR Methods. Primers used for qPCR.

Locus	Primer1	Primer2
parS-355	taattcatcatcgcgctcaa	aatgccgattacgagtttgc
yyaD	cttgcgatttttgcttctcc	acatcaccatacgtggacga
parS-359	aaaaagtgattgcggagcag	agaaccgcatctttcacagg
dnaA	gatcaatcggggaaagtgtg	gtagggcctgtggatttgtg
dnaN	gaattccttcaggccattga	gatttctggcgaattggaag
rplC	ttgacgacaagcgtgaaaag	ttcatacgcatccatttcca
yocGH	tccatatcctcgctcctacg	attctgctgatgtgcaatgg

Table S3, related to Figure 3. Enzymological parameters for Smc constructs with short coiled-coils or arm insertions.

Values are given as mean ± s.d. of best-fit values obtained from the indicated number of replicate experiments.

Construct	v_{max}	$K_{0.5}$	n	Replicates	Plasmid
WT	16.2 ± 2.1	0.48 ± 0.13	1.047 ± 0.092	6	pSG1497
CC296	17.21 ± 0.72	0.0537 ± 0.0060	1.608 ± 0.082	4	pSG2914
CC293	16.6 ± 3.4	0.0460 ± 0.0075	1.74 ± 0.10	4	pSG2915
CC257	19.3 ± 1.3	0.137 ± 0.01	1.345 ± 0.059	4	pSG2916
CC253	18.5 ± 1.4	0.274 ± 0.038	1.145 ± 0.059	4	pSG2917
CC296 (K37I)	n.d.	n.d.	n.d.	4	pSG2920
CC293 (K37I)	n.d.	n.d.	n.d.	4	pSG2921
394Ω	21.22 ± 0.70	0.1189 ± 0.0023	1.347 ± 0.089	2	pSG2965
479Ω	17.86 ± 0.64	0.1001 ± 0.0025	1.4394 ± 0.0090	2	pSG2966

Tables S4, S5 and S6 are available as separate files.

Table S4, related to Figure 1. Prediction of coiled coil length in natural SMC sequences.

Table S5, related to Figure 2. Construction and viability of shortened and elongated Smc variants based on Smc, *BsSp* Smc and Smc(Zh).

Table S6, related to Figure 6. Transposon insertion screen: Functional Smc proteins with peptide insertions.
