# Supplementary material for: Tuned SMC Arms Drive Chromosomal Loading of Prokaryotic Condensin
Source: Mol Cell. 2017 Mar 2;65(5):861–872.e9. doi: 10.1016/j.molcel.2017.01.026 (PMC5344682; doi:10.1016/j.molcel.2017.01.026)
Supplement: Document S2. Article plus Supplemental Information [file mmc5.pdf]

Molecular Cell

Article

Tuned SMC Arms Drive Chromosomal Loading of Prokaryotic Condensin

Graphical Abstract

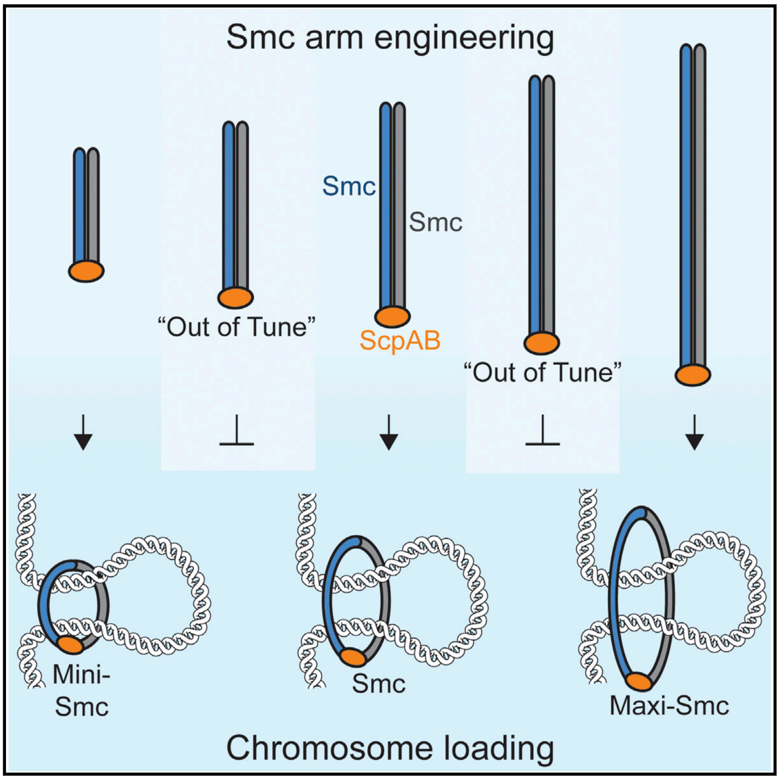

Authors

Frank Bürmann, Alrun Basfeld, Roberto Vazquez Nunez, Marie-Laure Diebold-Durand, Larissa Wilhelm, Stephan Gruber

Correspondence

stephan.gruber@unil.ch

In Brief

By engineering a series of Smc proteins with shorter or longer coiled-coil arms, Bürmann et al. elucidate a critical role for the arms' super-helical nature. Improper arms support chromosomal targeting but fail to link ATP hydrolysis to chromosomal loading. Smc arms are proposed to implement force transmission upon nucleotide hydrolysis.

Highlights

- Short and long but not intermediate-length Smc coiled-coil arms are functional
- Smc complexes with improper arms accumulate at chromosomal loading sites
- Smc arms are functional units linking ATP hydrolysis to an essential DNA transaction
- Pro- and eukaryotic SMC sequences reflect similar periodic length constraints

Bürmann et al., 2017, Molecular Cell *65*, 861–872
March 2, 2017 © 2017 The Author(s). Published by Elsevier Inc.
http://dx.doi.org/10.1016/j.molcel.2017.01.026

Molecular Cell
Article

CellPress

Tuned SMC Arms Drive Chromosomal Loading of Prokaryotic Condensin

Frank Bürmann,[1] Alrun Basfeld,[1] Roberto Vazquez Nunez,[2] Marie-Laure Diebold-Durand,[1] Larissa Wilhelm,[1] and Stephan Gruber[1,2,3,*]
[1]Max Planck Institute of Biochemistry, Am Klopferspitz 18, 82152 Martinsried, Germany
[2]Department of Fundamental Microbiology, University of Lausanne, Bâtiment Biophore, 1015 Lausanne, Switzerland
[3]Lead Contact
*Correspondence: stephan.gruber@unil.ch
http://dx.doi.org/10.1016/j.molcel.2017.01.026

SUMMARY

SMC proteins support vital cellular processes in all domains of life by organizing chromosomal DNA. They are composed of ATPase "head" and "hinge" dimerization domains and a connecting coiled-coil "arm." Binding to a kleisin subunit creates a closed tripartite ring, whose ∼47-nm-long SMC arms act as barrier for DNA entrapment. Here, we uncover another, more active function of the bacterial Smc arm. Using high-throughput genetic engineering, we resized the arm in the range of 6–60 nm and found that it was functional only in specific length regimes following a periodic pattern. Natural SMC sequences reflect these length constraints. Mutants with improper arm length or peptide insertions in the arm efficiently target chromosomal loading sites and hydrolyze ATP but fail to use ATP hydrolysis for relocation onto flanking DNA. We propose that SMC arms implement force transmission upon nucleotide hydrolysis to mediate DNA capture or loop extrusion.

INTRODUCTION

SMC protein complexes govern genome maintenance by controlling the 3D organization of chromosomes in interphase and during cell division, the cohesion and disjunction of sister chromatids, and the repair of DNA breaks. They also play roles in establishing patterns of gene expression during development and in disease (Hirano, 2016; Jeppsson et al., 2014; Merkenschlager and Nora, 2016; Peters and Nishiyama, 2012). All these functions are in all likelihood based on the co-entrapment of DNA double helices within the circumference of an SMC ring (Gligoris et al., 2014). However, two immediate questions remain unresolved: How does an SMC ring capture chromosomal DNA, and how does SMC choose suitable pairs of DNA segments for co-entrapment over inappropriate ones? Answering these key questions will require a detailed understanding of the chromosomal loading processes.

SMC rings are formed by a dimer of SMC proteins (in *Bacillus subtilis* [*Bs*], a Smc homodimer) and a single kleisin subunit (ScpA in *Bs*). The SMC proteins are composed of an ATPase "head" and a "hinge" dimerization domain and a long connecting coiled-coil "arm." The kleisin bridges the SMC heads to form a complex with circular topology (Bürmann et al., 2013; Gruber et al., 2003). SMC-kleisin rings associate with two Kite subunits (a homodimer of ScpB in *Bs*) or two Hawk subunits (Haering and Gruber, 2016; Palecek and Gruber, 2015; Wells et al., 2017) (Figure 1A). Additional factors are involved in the targeting and chromosomal loading of a given SMC complex. In *B. subtilis*, ParB/*parS* acts as loader for Smc-ScpAB by recruiting an Smc ATPase-cycle intermediate to the replication origin region (Gruber and Errington, 2009; Minnen et al., 2011; Sullivan et al., 2009; Wilhelm et al., 2015). Upon ATP hydrolysis, Smc-ScpAB relocates from *parS* loading sites to distant regions of the chromosome, conceivably in a DNA loop extrusion reaction (Gruber, 2014; Minnen et al., 2016; Wang et al., 2015). By co-aligning the two arms of the chromosome, Smc-ScpAB, with the help of its chromosomal loader ParB/*parS*, determines the global fold of the bacterial chromosome (Le et al., 2013; Marbouty et al., 2015; Umbarger et al., 2011; Wang et al., 2015). Consistent with the notion that related processes might organize chromosomes in eukaryotes, the cohesin SMC complex is known to relocate upon ATP hydrolysis from its centromeric loading sites onto flanking chromosome arm sequences in yeast (Hu et al., 2011).

All globular parts of the bacterial Smc-ScpAB complex are essential for its activity. Removal or partial dissociation of ScpAB renders the complex non-functional (Bürmann et al., 2013; Kamada et al., 2013; Mascarenhas et al., 2002; Soppa et al., 2002). Abrogation of dimerization at the Smc hinge domain results in non-functional protein (Bürmann et al., 2013; Hirano and Hirano, 2002; Minnen et al., 2016), and inactivation of the ATPase head is likewise detrimental (Bürmann et al., 2013; Mascarenhas et al., 2005; Minnen et al., 2016; Schwartz and Shapiro, 2011). Similar statements are valid for many other if not all SMC complexes.

In contrast to the globular parts, the functional importance of the SMC coiled-coil arm is less clear. Artificial opening of cohesin or condensin rings by proteolytic cleavage of their arms renders these complexes non-functional and releases them from DNA (Cuylen et al., 2011; Gruber et al., 2003; Ivanov and Nasmyth, 2005). Other than being passive barriers for entrapped DNA, it is not clear whether the arms have an active role in any biochemical mechanism of SMC complexes such as DNA capture or loop extrusion. However, point mutations in the coiled coils of cohesin SMC subunits have been identified in several Cornelia de Lange syndrome patients (Mannini et al., 2013; Orgil

This is an open access article under the CC BY license (http://creativecommons.org/licenses/by/4.0/).

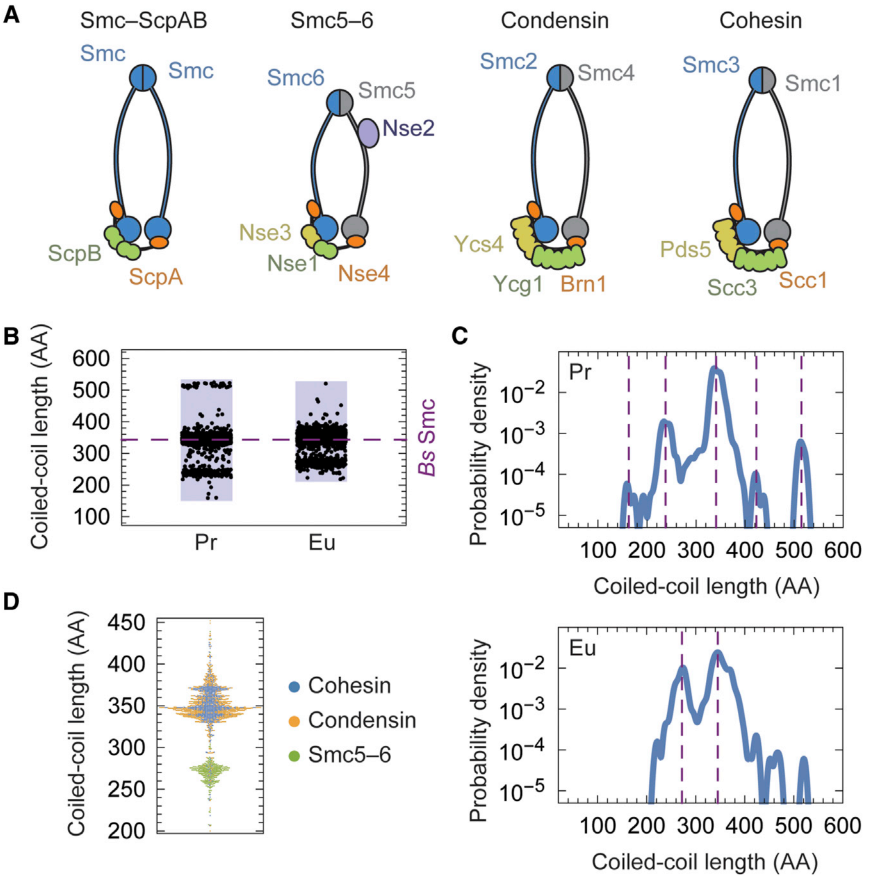

Figure 1. Coiled-Coil Length Distributions of SMC Proteins

(A) Subunit composition of SMC-kleisin complexes.

(B) Coiled-coil length distribution of prokaryotic (Pr; n = 3,337) and eukaryotic (Eu; n = 1,659) SMC sequences. Arm lengths were estimated on the basis of multiple sequence alignments. The dashed line indicates the coiled-coil length of *Bs* Smc.

(C) Kernel density estimates for data shown in (B). Dashed lines indicate positions of prominent modes.

(D) Arm length distribution for eukaryotic SMC sequences classified by type of complex.

See also Figure S1.

et al., 2016), consistent with the notion of additional functions for the SMC arms.

Here, we set out to delineate functional requirements for the arm in the biological activity of Smc-ScpAB. On the basis of large-scale protein engineering approaches involving a systematic change in the length of the arm and the random insertion of peptides, we have uncovered an unanticipated role that goes beyond its proposed function as a DNA barrier. The novel activity is tightly coupled to the ATPase cycle of Smc and requires an arm length matching a periodic pattern as well as the mechanical integrity of the arm. We find that disruptions in arm length or rigidity result in the aberrant accumulation of Smc proteins at the *parS* loading sites. Our data uncover a critical step of the chromosomal loading process and suggest that SMC arms mediate an essential long-distance DNA transaction driven by the SMC ATPase. We propose that the arm mediates the opening of a DNA entry gate during DNA entrapment or the active extrusion of DNA loops.

RESULTS

A Multimodal Distribution of SMC Arm Lengths

The sequences and structures of the globular SMC core domains and their interfaces are highly conserved throughout the phylogenetic tree (Bürmann et al., 2013; Gligoris et al., 2014; Griese et al., 2010; Haering et al., 2002, 2004; Nolivos and Sherratt, 2014; Woo et al., 2009). The SMC coiled-coil arm, in contrast, shows much less conservation at the sequence level (with the exception of cohesin in animals), and little is known about its structure (White and Erickson, 2006, 2009). Interestingly, however, its overall length is very similar in *Bs* Smc, condensin Smc2 and Smc4, and cohesin Smc1 and Smc3, apparently indicating strict evolutionary conservation of arm length.

To gain a more comprehensive view on the distribution of arm lengths in SMC proteins, we used an HHsenser-based pipeline to aggregate large sets of diverged SMC sequences (Söding et al., 2006) (Figure S1). For each sequence, we identified the positions of head and hinge regions by sequence alignment and used the mean length of the interlinking segments as an estimator for coiled-coil length. Surprisingly, we obtained a clearly multimodal length distribution both for prokaryotic and for eukaryotic sequences (Figures 1B and 1C). The kernel density estimate for prokaryotic Smc showed three major modes at 238, 341, and 515 amino acids (AA), respectively, and possibly two minor ones at 163 and 423 AA. Interestingly, albeit having a less well defined distribution, eukaryotic sequences generated two pronounced modes at lengths of 272 and 345 AA. The first one was produced predominantly by Smc5/6 sequences, whereas the second one was mostly generated by cohesin's Smc1/3 and condensin's Smc2/4 (Figure 1D). Puzzlingly, it appears that SMC arms underlie an evolutionary constraint that permits specific lengths in steps of ~100 AA but largely disfavors intermediate lengths. We note that the real length distributions might be even sharper than our estimates, because our method does not account for insertions of non-coiled-coil regions in the arm.

Periodic Length Constraints on the Smc Coiled Coil

To identify the functional basis of the length conservation, we systematically resized the coiled-coil arm of *Bs* Smc. On the basis of available disulfide register mapping and structural information (Bürmann et al., 2013; Minnen et al., 2016; Soh et al., 2015; Waldman et al., 2015), we designed a series of 258 successively

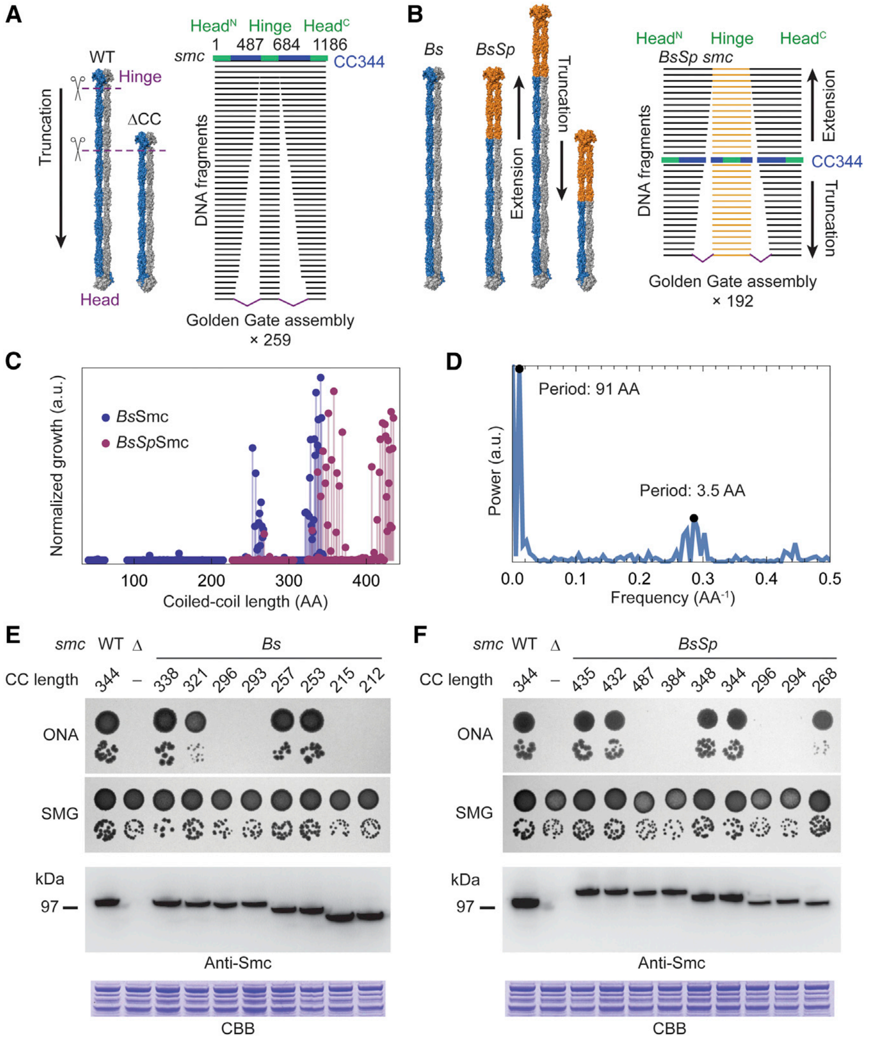

Figure 2. High-Throughput Screens for Functionally Resized Smc

(A) Strategy for an arm truncation screen. Smc arms were shortened by grafting the hinge and a short stretch of hinge-proximal coiled coil onto a shortened head-proximal part (left). An arrow illustrates the tested size-range. Shortened alleles were assembled by a Golden Gate approach (right).

(B) Strategy for an arm extension screen. Smc arms were either extended or shortened by resizing the *Bs* part of a functional chimeric *Bs*/*S. pneumoniae* (*BsSp*) protein. As in (A).

(C) Viability of strains with resized Smc arms. Modified *smc* alleles were transformed into *smc*-null cells for allelic replacement at the endogenous locus. Transformation mixtures were plated on ONA with antibiotics, and growth was assessed by the total area of bacterial colonies per plate. Truncation and extension screen were performed independently and normalized to their respective 95% growth quantile.

(D) Power spectrum of data shown in (C). The periods of the major peaks are indicated.

(E) Dilution spotting of strains with short *smc* alleles. Strains were constructed on SMG in the absence of selection pressure for *smc* function. Strains were spotted either on rich (ONA) or minimal (SMG) medium. Expression of the engineered alleles was probed by western blotting using polyclonal antibodies raised against full-length Smc. Note that modification of the Smc protein possibly removes some of the epitopes. Coomassie staining of extracts run on a separate SDS-PAGE gel is shown as a loading control. CBB, Coomassie Brilliant Blue; CC, coiled coil.

(F) Dilution spotting of strains with long *smc* alleles. As in (E).

See also Figure S2.

shortened Smc constructs, covering most of the coiled coil at amino-acid resolution (Figure 2A). We then used a Golden Gate assembly driven allelic replacement strategy to regenerate the endogenous locus of a *smc* deletion strain with the synthetic variants (Figure S2A). Transformation mixtures were plated on Oxoid nutrient agar (ONA) solid medium, a condition that is lethal for the parental *smc* null strain, and plates were imaged after 36 hr. Bacterial colonies were detected, and the ability of the corresponding constructs to support growth on rich medium was assessed by using the total colony area per plate as a proxy.

Starting from the wild-type arm length of 344 AA, we observed a gradual loss of function down to a length of 321 AA (Figure S2B). This was followed by a large region depleted of functional constructs. However, colonies reappeared at arm lengths between 267 and 253 AA, close to a major mode of the length distribution obtained by sequence analysis (Figures 1C and S2B). Next, we restored the shortest functional construct to full-length size by replacing its hinge domain with the hinge domain and ~100 AA hinge-proximal coiled coil of *Streptococcus pneumoniae* (*Sp*) Smc (Figures S2C–S2E). This chimeric protein, termed *BsSp*Smc, was then used to extend the SMC arm beyond its natural length (Figures 2B and S2B). By this approach, we obtained functional constructs in the arm-length regions of 330–373 AA and 407–435 AA, separated by a gap of non-functional constructs. Strikingly, a clear multimodal distribution of viability became apparent in the merged data set of the shortening and the extension screen (Figures 2C and 2D), which we confirmed by strain reconstruction without selecting for Smc function (Figures 2E and 2F).

We then used Fourier analysis to extract underlying periodicities in the viability data set (Figure 2D). Intriguingly, the power spectrum showed two prominent peaks: one major peak at a period of 91 AA, close to the super-helical coiled-coil period of ~99 AA, and a minor one at a period of 3.5 AA, which is the α-helical period in coiled coils (Truebestein and Leonard, 2016). Thus, our genetic data appear to faithfully reflect the 3D structure of the arm and links it directly to a biologically relevant output. We conclude that the evolutionary length distribution of SMC sequences has a functional basis and that this function is largely determined by the super-helical structure of their coiled coil.

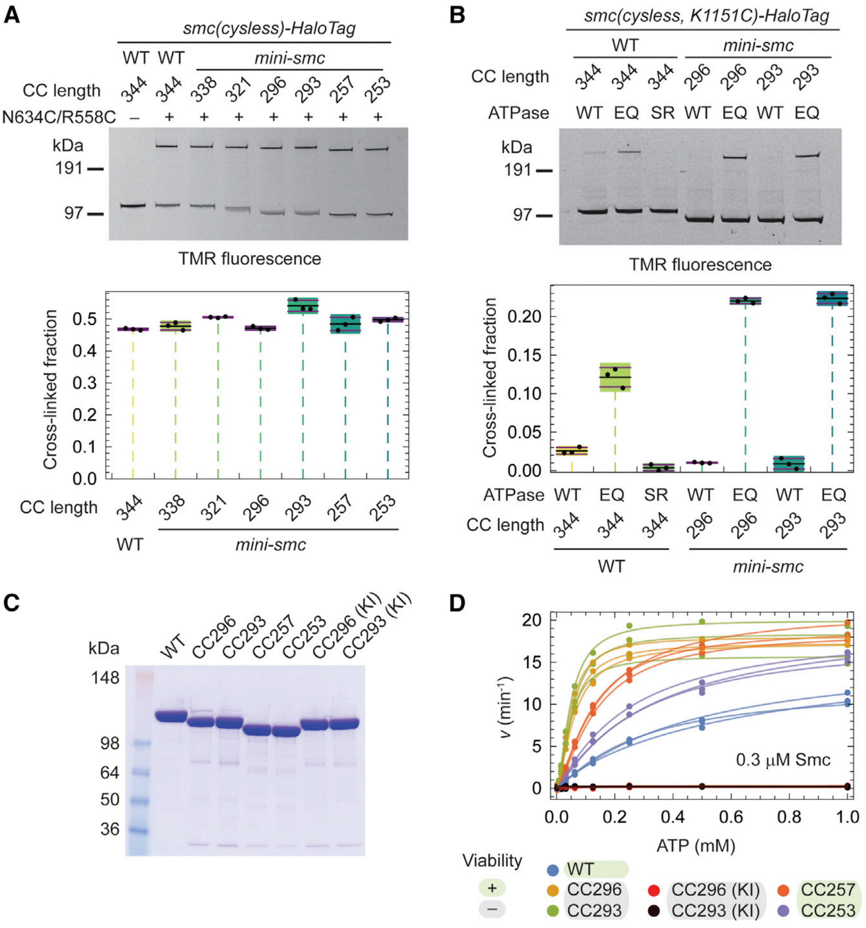

Figure 3. Dimerization and ATPase Activity of Mini-Smc Proteins

(A) In vivo site-specific crosslinking of Mini-Smc variants at the hinge interface (see also Figure S2B). In-gel fluorescence after BMOE crosslinking of strains containing cysless Smc-HaloTag variants (top) and quantification thereof (bottom) is shown. Crosslinking was performed in three separate reactions. Colored boxes indicate 95% credible intervals, horizontal lines indicate mean and SD of the data.

(B) Head engagement levels in Mini-Smc proteins monitored by in vivo site-specific crosslinking at the reporter residue K1151C (see also Figure S3C) (Lammens et al., 2004; Minnen et al., 2016). The SR mutation blocks head engagement, and the EQ mutation blocks ATP hydrolysis (Figure S3A). As in (A).

(C) Purification of Smc variants. Purified fractions were analyzed by SDS-PAGE and Coomassie staining. KI, Smc ATP-binding mutation K37I.

(D) Steady-state ATPase activity of purified Smc variants at 0.3 μM protein and variable ATP concentration. Activity was determined by a coupled enzyme assay and data were fitted by the Hill model (see also Table S3). Data and fits for three replicates are shown.

See also Figure S3.

Mini-Smc Dimerization and ATPase Activity

Aiming to assess whether alterations in the coiled-coil sequences lead to gross protein folding defects, we characterized the dimerization and enzymatic activity of selected Mini-Smc constructs. We performed site-specific cysteine crosslinking experiments in vivo and found that dimerization at the hinge domain was normal in the six tested Mini-Smc proteins (Figures 3A and S3B). Likewise, the Smc head domains engaged robustly in the presence of a mutation blocking ATP hydrolysis (E1118Q [EQ]), whereas head engagement was hardly detectable in the absence of this mutation, as observed with full-length Smc (Minnen et al., 2016) (Figures 3B and S3C; see Figure S3A for an overview of the SMC ATP hydrolysis cycle). Curiously, the efficiency of head engagement in Smc(EQ) proteins increased somewhat as the coiled coil was shortened (Figures 3B and S3D), consistent with our previous proposition that the formation of a rod by close juxtaposition of the two Smc coiled coils hinders head engagement (Minnen et al., 2016; Soh et al., 2015).

We then purified Mini-Smc proteins to measure their ability to hydrolyze ATP in vitro (Figure 3C). All tested functional and non-functional Mini-Smc proteins hydrolyzed ATP slightly faster than wild-type Smc at saturating ATP concentrations (higher v_{max}) and substantially faster at sub-saturating ATP concentrations (lower $K_{0.5}$) (Figures 3D and S3E; Table S3). These findings are consistent with the enhancement of head-engagement detected by crosslinking. Curiously, the non-functional, intermediate-length Mini-Smc proteins displayed the highest apparent affinity for ATP (Figure 3D; Table S3). The observed ATPase rates likely originate from isolated Smc dimers (and not from inter-dimer collisions), as they are largely independent from protein concentration (Figure S3F).

Taken together, we conclude that the engineered non-functional Smc variants do not display gross folding defects and fail to support viability for more specific reasons.

The Coiled Coil Determines Chromosomal Loading of Smc

Recruitment of Smc-ScpAB to the chromosome has been linked to a conformational change in the coiled-coil arm (Minnen et al., 2016; Soh et al., 2015). We therefore investigated whether targeting of Smc-ScpAB to the chromosome was perturbed in complexes containing Smc variants with shorter coiled coils. To this end, cells were grown in minimal medium (SMG) and analyzed by α-ScpB chromatin immunoprecipitation coupled to deep sequencing (ChIP-seq). Surprisingly, ChIP-seq profiles of non-functional Mini-Smc complexes revealed a pronounced enrichment at *parS* sites compared with other chromosomal locations, whereas wild-type protein was strongly enriched at the origin of replication (*oriC*), but less so at *parS* (Figures 4A and S4A). The relative enrichment of Mini-Smc proteins at and near *parS* sites and the relative depletion from *oriC* and chromosome arms compared with wild-type became especially apparent in ratiometric comparisons of the ChIP-seq profiles (Figures 4A and S4A, bottom). This suggests that although Mini-Smc

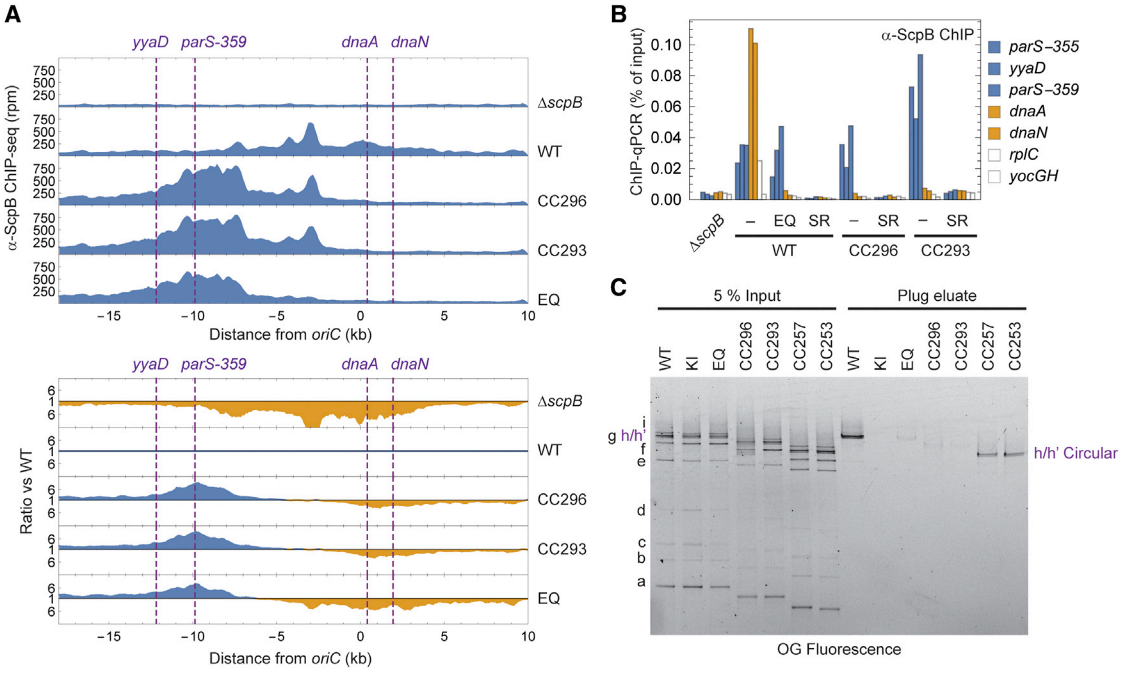

Figure 4. Chromosomal Loading of Smc-ScpAB Containing Mini-Smc Proteins

(A) ChIP-seq profiles at the *oriC* region. ChIP was performed with an antiserum raised against ScpB. Normalized counts in reads per million (rpm) are plotted against the distance from *oriC* (top). The bottom graph shows the ratiometric analysis against the wild-type profile. For each bin, normalized counts were compared with the respective wild-type value. The higher value was divided by the lower. For bins where the mutant value was greater than or equal to the wild-type value, the ratio was plotted above the genome coordinate axis (blue) and below the axis otherwise (orange). EQ, Smc(E1118Q).

(B) ChIP-qPCR against ScpB for *mini-smc* strains containing an ATPase mutation that prevents head engagement (SR, S1090R). Loci close to *parS* sites are colored in blue, loci close to the replication origin are orange, and chromosomal arm positions are white (see Figure 4A, top).

(C) Chromosome entrapment assay for strains containing Mini-Smc complexes. Smc-ScpAB complexes containing Smc-HaloTag variants were site-specifically cross-linked at hinge and ScpA-Smc interfaces and conjugated to a HaloTag-OregonGreen (OG) substrate. Intact chromosomes were isolated in agarose plugs, and proteins were extracted under denaturing conditions. Smc-HaloTag species retained in the plug were resolved by SDS-PAGE and detected by in-gel fluorescence. Species a–g and i are linear, species h/h′ are circular.

See also Figure S4.

constructs were able to target the loading sites, they failed to redistribute to adjacent loci.

Interestingly, this localization phenotype is similar to the EQ mutant, which in contrast to the Mini-Smc proteins is blocked in ATP hydrolysis (Hirano and Hirano, 2004; Minnen et al., 2016) (Figures 4A and S4A). We confirmed these findings by ChIP coupled to quantitative PCR (qPCR), which also showed that the extent of redistribution correlated well with the ability of Mini-Smc proteins to support fast growth (Figure S4B). Importantly, chromosomal recruitment was still dependent on Smc head engagement, because the engagement-blocking mutation S1090R (SR) abrogated localization (Hirano et al., 2001; Minnen et al., 2016) (Figure 4B). However, continuous head engagement seems dispensable for association with the loading sites at least in Mini-Smc proteins, which engage heads only transiently (Figure 3B). Together, these findings imply that Mini-Smc proteins can successfully complete their ATPase cycle but fail to couple ATP hydrolysis to an essential activity that is accompanied by re-localization on the chromosome.

In addition to triggering chromosomal redistribution, the Smc ATP hydrolysis activity has been linked to the capture of DNA inside the Smc-ScpAB ring. We therefore tested for the association of Mini-Smc variants with chromosomal DNA by the chromosome entrapment assay. This is based on the isolation of intact chromosomal DNA in agarose plugs and the co-purification of Smc-ScpA species that have been site-specifically crosslinked into covalent rings (Wilhelm et al., 2015). All four tested Mini-Smc proteins associated normally with the kleisin ScpA as judged by their crosslinking patterns (Figure 4C). Although the circular species derived from functional Mini-Smc proteins were retained during chromosome isolation, non-functional Mini-Smc rings were almost completely extracted from the chromosome plugs, similar to covalent rings obtained for Smc(EQ). This shows that non-functional Mini-Smc proteins largely fail to entrap chromosomal DNA, possibly because they are directly blocked in an entrapment reaction (e.g., topological loading or non-topological extrusion of large loops). Alternatively, they might be blocked in an upstream or downstream rate-limiting process (e.g., clearance of loading sites).

In summary, both known ATP hydrolysis-dependent activities of Smc are specifically lost in the non-functional Mini-Smc proteins (i.e., redistribution from chromosomal loading sites and the entrapment of DNA). Thus, Smc proteins with illegitimate

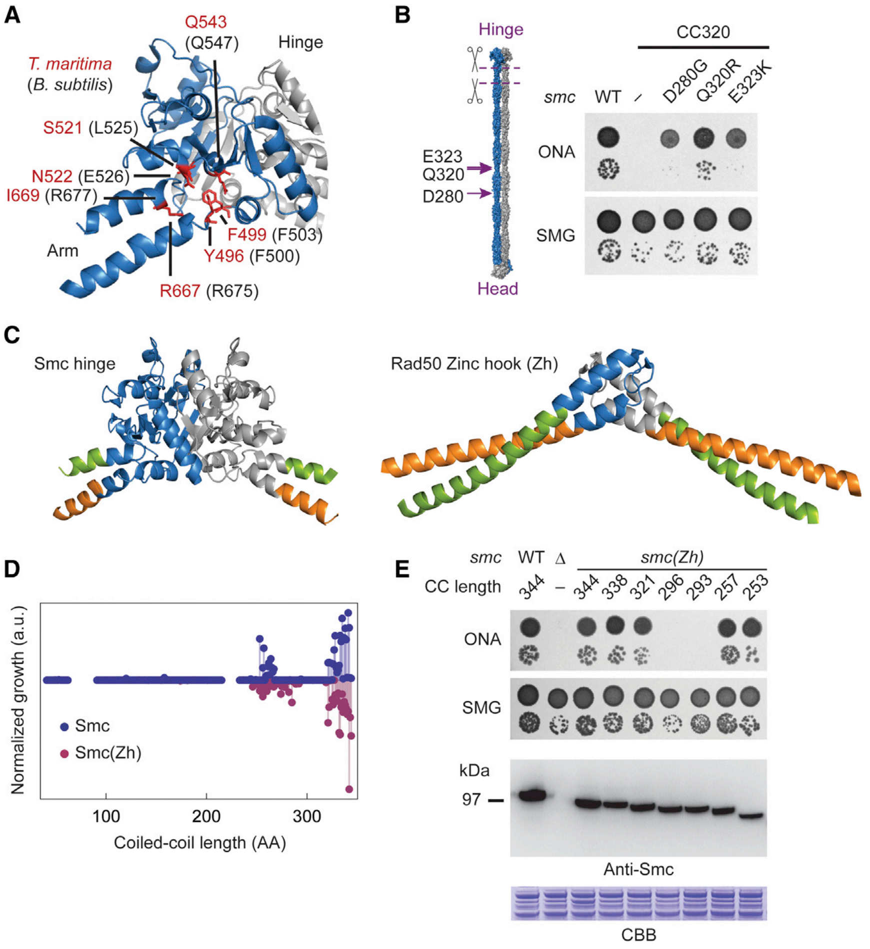

Figure 5. Suppressor Mutagenesis and Hinge Replacement of Mini-Smc Variants

(A) Suppressor mutations mapped onto the crystal structure of the *T. maritima* (*Tm*) hinge domain (Protein Data Bank [PDB]: 1GXL). *Bs* residues are indicated in black, *Tm* homologs are indicated in red.

(B) Mutations in the arm suppress lethality of the CC320 *mini-smc* allele. The cartoon illustrates the position of the suppressor mutations (left). The panel on the right shows spot dilutions as in Figure 2D.

(C) Comparison of open conformations of the SMC hinge (left; PDB: 1GXL) and the Rad50 Zinc hook (right; PDB: 1L8D). The N-terminal coiled-coil strands are colored green, and the C-terminal strands are colored orange. The part that was substituted to construct a functional Smc(Zh) chimera is shown in blue and gray, respectively.

(D) Coiled-coil truncation screen of the Smc(Zh) protein. Data are compared with the arm shortening experiment shown in Figure 2C. The growth axis for Smc(Zh) has been inverted for clarity. As in Figure 2C.

(E) Spot dilutions and western blot analysis of Smc(Zh) variants with short coiled coils. As in Figure 2E.

See also Figure S5.

lengths of coiled coil are unable to couple ATP hydrolysis to an essential DNA transaction on the chromosome.

An Unrelated Dimerization Domain Supports Smc Function

The above results suggest that the Smc coiled coil acts as a functional unit with considerable rigidity. A change in the length of the coiled-coil arm might thus alter the phase relationship between its ends; that is, it will modify the orientation of the hinge with respect to the head (Figure S5A). If so, then locally relaxing Smc rigidity, for example by introducing point mutations, might compensate for the shortening of the Smc coiled coil. To test this, we applied error-prone PCR to screen all non-functional constructs in the length region of 270–320 AA (named CC270–CC320) for suppressor mutations in the Smc hinge domain and 12 AA of the associated coiled coil. However, good suppressor mutations were identified only for a limited number of constructs, all harboring a coiled coil with borderline length (Figures S5B and S5C). Most of the mutations mapped to a conserved hydrophobic pocket that appears to fix the hinge onto the arm (Figure 5A) (Haering et al., 2002; Soh et al., 2015). We next screened the borderline length construct CC320 for suppressor mutations in other parts of Smc (except for the N-terminal head region). This yielded few additional suppressors located in the Smc coiled coil (Figure 5B). None of the isolated mutations, however, suppressed major coiled-coil length alterations. Perturbing Smc structure thus compensates for minor deviations in coiled-coil length only. These results provide support for the notion that a significant level of rigidity in the Smc coiled coil is functionally important. Intriguingly, however, the structural integrity of the coils/hinge junction appears less critical, because apparently disruptive point mutations are easily isolated in the respective part of the protein (Figure 5A).

With the aim to test more directly whether the hinge structure is crucial for Smc function, we next substituted the Smc hinge domain for the structurally unrelated Zinc-hook (Zh) dimerization domain of the SMC-like Rad50 protein from *Pyrococcus furiosus*. According to available structural information, the Zh and hinge domains connect differently to the corresponding coiled-coil arm (Figure 5C) (Hopfner et al., 2002). However, the Zh dimerization domain permitted apparently near-normal arm/arm association in a chimeric Smc(Zh) protein (Figure S5F). Strikingly, the Smc(Zh) protein also supported normal growth on nutrient rich medium. The fold of the dimerization domain in Smc is thus irrelevant for chromosome segregation in *Bacillus subtilis*.

We then truncated the arm of the Smc(Zh) construct to test for any changes in the constraints on arm length. To our surprise, we obtained a similar bimodal pattern as for the constructs with a wild-type Smc hinge (Figures 5D and 5E), possibly implying that a defined attachment of the coiled coil to the dimerization domain is not required for Smc function. More likely, however, the geometry of the attachment might be more similar in the

CellPress

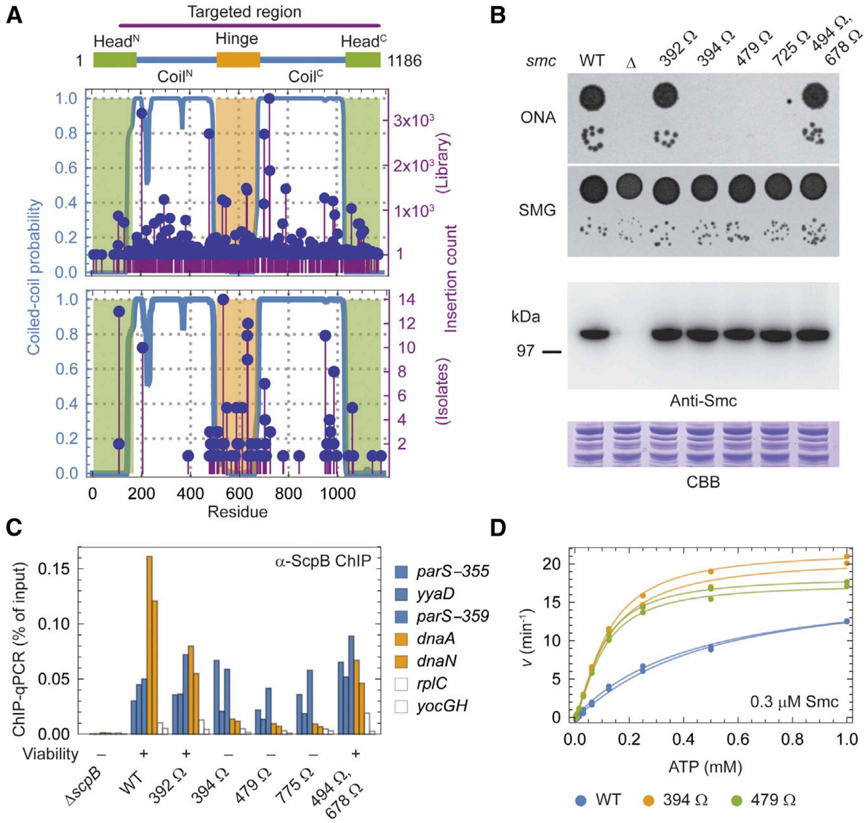

Figure 6. Transposon Screen for Functional Smc Variants Containing a Randomly Inserted Peptide

(A) Peptide insertion screen. The cartoon on top illustrates the region that was targeted by transposon mutagenesis of a *smc*-targeting construct. The obtained insertion library was characterized by deep sequencing, and reads containing the insert were selected. Insertion read counts for positions with at least one detected insertion are shown (top). After transformation of the library into a *smc*-null strain, viable clones isolated on ONA were characterized by Sanger sequencing. Counts of insert positions among viable isolates are shown (bottom). Green regions delineate the head domain, orange delineates the hinge region, and the blue graph indicates coiled-coil probability.

(B) Spot dilutions of strains with designed peptide insertions in the coiled-coil arm. As in Figure 2E.

(C) ChIP-qPCR against ScpB for strains containing peptide insertions in the Smc arm. Loci close to Smc loading sites are colored in blue, loci close to the replication origin are orange, and chromosomal arm positions are white (see Figure 4A).

(D) ATPase activity of non-functional Smc variants with peptide insertions in the coiled-coil arm. As in Figure 3D.

See also Figure S6.

families of Rad50 and SMC proteins than anticipated from available crystal structures (Figures 5C, S5F, and S5G).

Together, these findings demonstrate that the nature of the dimerization domain is surprisingly uncritical, and that the structure of the arm dominates the phenotype observed for Mini-Smc proteins.

The Integrity of the Smc Coiled Coil Is Critical for Chromosomal Loading

Apparently, the Smc arm couples ATP hydrolysis at the Smc heads to an essential chromosomal activity. Assuming a scenario in which the arm transmits information from the head to its distal end (or vice versa) (Hirano and Hirano, 2006; Minnen et al., 2016; Soh et al., 2015), its function might become compromised if such transmission was blocked by other means than altering its length. We reasoned that this might be achieved by inserting a flexible peptide into the transmission pathway. Therefore, from a set of Smc proteins with a peptide insertion at random positions, all constructs disrupting the force transmission pathway might be depleted after selection for Smc function. Following this strategy, we isolated functional Smc variants with an insertion of a 14-AA-long peptide at a random position. Briefly, we used in vitro transposon mutagenesis and sub-cloning to generate a library of double-crossover gene-targeting constructs that contained short sequences inserted into the *smc* open reading frame. The library was characterized by deep sequencing (Figures 6A and S6A) and was subsequently transformed into a *smc* deletion strain for allelic replacement. We isolated 190 viable insert-containing clones on ONA and characterized them by Sanger sequencing. Many of the recovered alleles contained insertions in the hinge domain, which mostly mapped to loops or surface exposed structural elements (Figure S6B). Intriguingly, the set of viable isolates was considerably depleted of inserts in the coiled coil (Figure 6A). Whereas the arm accommodated 70% of inserts in the input library, this fraction was reduced to 42% in the set of functional isolates ($p < 0.001$ by approximate permutation test). Furthermore, the distribution of coiled-coil inserts among the functional proteins was highly non-uniform, with hotspots close to the hinge and at the head- and hinge-proximal coiled-coil breaks, respectively (Minnen et al., 2016; Waldman et al., 2015). Insertions in the N-terminal helix were particularly rarely recovered.

We corroborated our findings by targeted strain construction in the absence of selection pressure for Smc function, whereby many of the designed mutants displayed a lethal phenotype on ONA albeit producing wild-type levels of protein (Figure 6B). It appears that Smc can be readily modified in or at the hinge domain, but not in most parts of its coiled coil, consistent with the notion that the arm might act as a mechanical device for information transmission. Excitingly, Smc-ScpAB complexes containing Smc variants with peptide insertions in their arm were impaired in chromosomal redistribution, similar to complexes containing Mini-Smc proteins (Figures 6C and S4B). The extent of this phenotype correlated well with viability. Moreover, Smc proteins with peptide insertions are functional ATPases with slightly higher v_{max} and considerably lower $K_{0.5}$ parameters

compared with wild-type protein, similar to the Mini-Smc proteins (Figure 6D; Table S3). Taken together, we conclude that the full coiled-coil arm is intimately involved in a chromosomal DNA transaction during ATP hydrolysis and that this activity is absolutely essential for Smc function.

DISCUSSION

The SMC Coiled Coil as a Functional Unit

The DNA entrapment model has been widely used to explain the biological activities of SMC-kleisin rings. Naturally, DNA entrapment requires a barrier that prevents DNA escape. Although the precise location of DNA within SMC complexes is unknown, the arms likely act as such a barrier because they make up a large part of the ring circumference. This notion is supported by the finding that artificial proteolytic cleavage of the coiled coil releases both cohesin and condensin from chromatin (Cuylen et al., 2011; Gruber et al., 2003). If preventing DNA loss from the complex would sufficiently describe the function of the SMC arms, then constraints on their structure are expected to be low: physical integrity and a minimum length to accommodate the substrate should suffice. Other properties such as rigidity would probably be unconstrained or even disfavored.

Electron microscopy, small-angle X-ray scattering, crosslinking/mass spectrometry, and crystallographic experiments for several SMC complexes suggest that the arms are rigid at least over a considerable distance (Anderson et al., 2002; Barysz et al., 2015; Hirano et al., 2001; Huis in 't Veld et al., 2014; Soh et al., 2015). In contrast, a recent study of Smc2–4 heterodimers in the atomic force microscope has proposed a persistence length of about 5 nm for the yeast condensin coiled coil (Eeftens et al., 2016). Compared with a continuous coiled coil with an expected persistence length of about 150 nm, this is surprisingly flexible (Wolgemuth and Sun, 2006) and would suggest that the arms of condensin might rather act as passive domain linkers than as mediators of a biochemical activity. Here, we present functional evidence that this is not the case for the coiled coil of Smc-ScpAB. First, the arm of *B. subtilis* Smc tolerates flexible insertions in few positions only, implying that it acts as a functional unit rather than a chain of loosely connected coiled-coil segments. Second, a long-distance geometrical relationship within the arm, determined by its super-helical structure, appears crucial for Smc function. This property is reflected in the length distributions of both prokaryotic and eukaryotic SMC sequences and probably also in those of the more distantly related MukB, MksB and Rad50 proteins (Figure S1). We reason that the coiled-coil arm of bacterial Smc acts as a single functional unit and that this finding may generalize to many if not all types of SMC and SMC-like proteins. Consistent with this notion, the amino acid sequences of eukaryotic SMC coiled coils, particularly in cohesin, were found to be conserved well beyond the levels observed for spacer rods (White and Erickson, 2006).

Periodic Patterns in Coiled Coils

Coiled coils are formed by α helices with repetitive amino acid sequence patterns. A heptad repeat typically dominates at the fine level of coiled coil sequences, but non-canonical geometries with periods of, for example, 4, 11, 15, or 18 residues do exist (Gruber and Lupas, 2003; Lupas and Gruber, 2005; Truebestein and Leonard, 2016). Interfering with the heptad register disturbs or eliminates protein function whenever a precise local structure is important. For example, the transcription factor Gcn4 tolerates 7-amino acid insertions between its DNA binding domain and the leucine zipper, while 2-, 4-, or 6-amino acid insertions misalign the two DNA binding domains in a given Gcn4 dimer and hinder DNA binding (Pu and Struhl, 1991). Similar observations have been made in several engineered histidine kinase dimers, in which extension or shortening of a coiled-coil domain linker changes the orientation of the signaling domain in a phase-dependent manner (Cochran and Kim, 1996; Möglich et al., 2009). In the case of Smc, we have also observed that locally breaking the heptad repeat interferes with protein function (Figure 2D), which is very likely caused by related effects. The requirement for a continuous heptad periodicity is particularly clear in Smc proteins that harbor a chimeric *BsSp* arm with a wild-type-like length (Table S5; Figure S2B, right).

Long-range periodicities in coiled-coil sequences have been defined in only a limited number of cases. For example, tropomyosin folds into a continuous coiled coil of about 280 residues comprising seven roughly equally sized repeat units (Barua, 2013). The units bind actin monomers within a filament and are aligned along the tropomyosin coiled-coil superhelix. Internal deletion of an entire repeat is tolerated. However, removal of half a repeat or one third of a repeat is interfering with actin binding and regulation, probably because of misalignment of the actin-binding sites (Hitchcock-DeGregori and Varnell, 1990). The 1,000-residue rod of myosin II contains a strong 28-repeat in charged residues, which probably promotes packing of myosin into ordered filaments (Decker and Kellermayer, 2008; McLachlan and Karn, 1982). Accordingly, 14 residues insertions or deletions alter the packing mode (Atkinson and Stewart, 1991). In these two examples, the long-range repeat pattern allows the association of the coiled coil with repetitive structures: the actin polymer and other myosin monomers, respectively. In case of Smc, however, no such interactions are known at the moment. Although the helical pitch of DNA and SMC arms are not compatible, Smc arms from different complexes could in principle pack into filaments, for example to drive a treadmilling reaction (Alipour and Marko, 2012). Alternatively, misaligned Smc arms might prevent the formation of a stable rod interface within the Smc dimer (Soh et al., 2015). However, we favor the idea that the arm serves a mechanical function, in addition to forming the dimer rod.

In Rad50 proteins, there is conformational crosstalk between the distantly located head and zinc hook dimerization domains, presumably mediated via the coiled-coil arms (Hohl et al., 2015). Artificially truncated versions of the yeast Rad50 protein are defective in genome maintenance, together underscoring the importance of the Rad50 arm in the repair of DNA (Hohl et al., 2011). Whether any periodic elements in the arm are critical, however, is unclear because only a handful of truncation constructs were tested. Systematic alterations of CC length might uncover many more examples of

CellPress

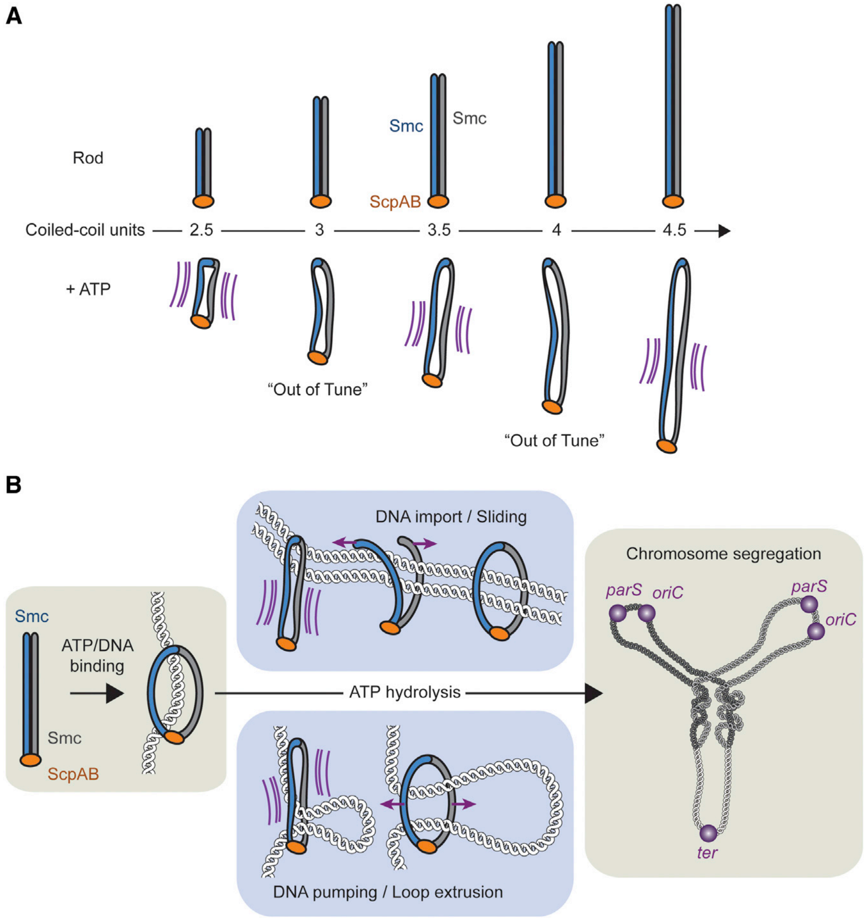

Figure 7. Models for the Role of the Coiled-Coil Arm during DNA Transactions of SMC

(A) Tentative model for the effect of arm length variation on Smc function. Proteins with a large offset in the super-helical phase of their coiled coils ("Out-of-Tune" complexes) react differently to mechanical strain induced during their ATPase cycle.

(B) Models for Smc arm function during chromosomal DNA transactions. After initial recruitment to the chromosome induced by ATP binding, the coiled-coil arms of Smc transduce mechanical energy to open a DNA entry gate (top middle) or directly act on DNA, for example during loop extrusion (bottom middle).

long-range patterns in coiled coil proteins with potentially novel functions.

Proper Arm Geometry Is Required for an ATPase-Driven DNA Transaction

Given the structural similarities of SMC complexes, it is conceivable that their biological activities are based on a considerably conserved biochemical mechanism. Consistently, cohesin, condensin and Smc-ScpAB each have been shown to entrap DNA within their ring structure (Cuylen et al., 2011; Gligoris et al., 2014; Wilhelm et al., 2015). From an abstract perspective, the chromosomal activity of SMC complexes may be partitioned into two phases: targeting and redistribution. Of those, targeting requires ATP-dependent head engagement, whereas redistribution also requires nucleotide hydrolysis (Hu et al., 2011; Minnen et al., 2016). Our findings now show that the coiled-coil arm mediates an essential DNA transaction after targeting (i.e., during the redistribution phase). This activity is in all likelihood directly coupled to nucleotide hydrolysis, because proteins with defective arms resemble the localization phenotype of the hydrolysis-deficient Smc(EQ) protein. We envision that the redistribution phase is composed of the active DNA entrapment process and another unknown process that leads to the actual disengagement from the loading site. The latter activity might be related to an active extrusion of DNA and require continuous ATP hydrolysis, or it might represent processive diffusion along the substrate, driven by external motors or thermal motion (Alipour and Marko, 2012; Goloborodko et al., 2016; Nasmyth, 2001). Thus, a proper geometry of the coiled-coil arm is directly required either for DNA entrapment or for a hypothetical ATP-driven movement along DNA, or for both. Alternatively, the coiled-coil arms may inhibit the Smc ATPase cycle (Figure 3D) to prevent ATPase driven unloading of Smc-ScpAB from chromosomes. ATP hydrolysis mediated unloading has been proposed recently for the related cohesin complex (Elbatsh et al., 2016; Huber et al., 2016). Resolving those exciting alternatives will be crucial for our understanding of SMC complexes, and will possibly require the establishment of single-molecule observations in a purified system.

The Arms as Force Transmitters during Chromosomal Loading

How might the SMC arm promote DNA loading? DNA capture by cohesin has been proposed to be mediated by the transient ATP hydrolysis-driven opening of the cohesin ring. Conceivably, mechanical communication between the head domains and the distal end of the coiled coil might promote opening of an entry gate, which has been suggested to be located at the Smc1/Smc3 hinge (Buheitel and Stemmann, 2013; Gruber et al., 2006) or the Smc3/Scc1 interface (Murayama and Uhlmann, 2015). We envision a scenario whereby the geometry of the SMC arm is tuned in such a way that it accommodates substantial strain upon head engagement and even more so during ATP hydrolysis and that this strain eventually dissipates by opening the DNA entry gate (Figure 7). Changing the coiled-coil length or flexibility might result in a geometry that can more easily accommodate such strain without opening the entry gate, and might thus uncouple gate opening from ATP hydrolysis.

If entry-gate opening also occurred in Smc-ScpAB, then this process must be feasible without direct contact between the hinge and other factors such as the ATPase head domain. This

is implied by our finding that the Smc hinge can be functionally substituted by the structurally unrelated Rad50 Zh domain. Although the Smc(Zh) protein does not contain a hinge, its activity still depends on a proper coiled-coil geometry (Figure 5D), indicating that opening of the DNA entry gate might be mainly mediated by the arms. A corollary is that such a mechanism would be remarkably robust, because it can tolerate different dimerization domains, flexible peptide insertions in the hinge-proximal coiled coil and substantial truncations thereof (Figures 2, 5, and 6).

The SMC Arms Promoting Chromosomal Relocation

Apart from allowing the topological capture of DNA, SMC arms may play a direct role in the relocation of SMC from loading sites. They could do so by actively extruding DNA or by enabling SMC diffusion along DNA driven by thermal motion or external motors. Dissolving the SMC rod (i.e., the state with associated arms) during DNA loading and simply re-forming this state upon ATP hydrolysis might hinder the passage of DNA tracking motors through the collapsed SMC complex (Stigler et al., 2016). The work of external motor proteins could thereby be harnessed to pull DNA through the complex. According to this hypothesis, formation of Smc rods should be defective in short, non-functional Smc proteins. However, our initial attempts based on cysteine crosslinking at few available positions failed to uncover an obvious correlation between the local organization of the Smc rod in Mini-Smc constructs and their ability to re-localize on the chromosome or promote growth (data not shown). The arms could also play a more active role during chromosomal redistribution. By re-forming Smc rods, they may for example push DNA from the head domains toward the hinge (or vice versa), similar to the action of a peristaltic pump (Figure 7). If so, then the phase shift in Mini-Smc proteins might disrupt the flow of DNA between the hinge and the head domains.

Altogether, we conclude that any future model for SMC activity needs to incorporate the coiled coil as a major functional component rather than a passive barrier and domain linker.

STAR★METHODS

Detailed methods are provided in the online version of this paper and include the following:

- KEY RESOURCES TABLE
- CONTACT FOR REAGENT AND RESOURCE SHARING
- EXPERIMENTAL MODEL AND SUBJECT DETAILS
  - *Bacillus subtilis* Strains and Growth
- METHOD DETAILS
  - Protein Sequence Analysis
  - High-throughput Allelic Replacement Screening
  - Suppressor Screening
  - Protein Purification and ATPase Activity Assay
  - Site-specific in vivo Cross-linking
  - Chromosome Entrapment Assay
  - Chromatin Immunoprecipitation
  - Transposon Insertion Screen
- QUANTIFICATION AND STATISTICAL ANALYSIS
  - Analysis of Cross-Linking Efficiencies
  - Fourier Analysis
  - Steady-State Enzyme Kinetics
  - Analysis of qPCR Data
  - Analysis of ChIP-seq Data
  - Analysis of Insertion Library Sequencing Data
  - Permutation Test
  - Kernel Density Estimation
- DATA AND SOFTWARE AVAILABILITY

SUPPLEMENTAL INFORMATION

Supplemental Information includes six figures and six tables and can be found with this article online at http://dx.doi.org/10.1016/j.molcel.2017.01.026.

AUTHOR CONTRIBUTIONS

A.B. performed the transposon insertion screen. M.-L.D.-D. performed disulfide mapping of the coiled-coil register. R.V.N. performed ATPase activity measurements. L.W. conducted the chromosome entrapment experiment. F.B. conducted all other experiments. F.B. and S.G. conceived experiments and prepared the manuscript.

ACKNOWLEDGMENTS

We are grateful to Kim Nasmyth for stimulating discussions at the outset of the project and for critical comments on the manuscript. We thank Anna Anchimiuk for technical help and Wolfgang Zachariae and Jürg Müller for sharing equipment and the sequencing facility at the Max Planck Institute for Plant Breeding Research for NGS library preparation and Illumina sequencing. A European Research Council Starting Grant (260853) to S.G., the Max Planck Institute of Biochemistry, Max Planck Society, and the University of Lausanne supported our research.

Received: October 24, 2016
Revised: December 23, 2016
Accepted: January 18, 2017
Published: February 23, 2017

REFERENCES

Alipour, E., and Marko, J.F. (2012). Self-organization of domain structures by DNA-loop-extruding enzymes. Nucleic Acids Res. *40*, 11202–11212.

Anderson, D.E., Losada, A., Erickson, H.P., and Hirano, T. (2002). Condensin and cohesin display different arm conformations with characteristic hinge angles. J. Cell Biol. *156*, 419–424.

Atkinson, S.J., and Stewart, M. (1991). Molecular basis of myosin assembly: coiled-coil interactions and the role of charge periodicities. J. Cell Sci. Suppl. *14*, 7–10.

Barua, B. (2013). Periodicities designed in the tropomyosin sequence and structure define its functions. BioArchitecture *3*, 51–56.

Barysz, H., Kim, J.H., Chen, Z.A., Hudson, D.F., Rappsilber, J., Gerloff, D.L., and Earnshaw, W.C. (2015). Three-dimensional topology of the SMC2/SMC4 subcomplex from chicken condensin I revealed by cross-linking and molecular modelling. Open Biol. *5*, 150005.

Buheitel, J., and Stemmann, O. (2013). Prophase pathway-dependent removal of cohesin from human chromosomes requires opening of the Smc3-Scc1 gate. EMBO J. *32*, 666–676.

Bürmann, F., Shin, H.C., Basquin, J., Soh, Y.M., Giménez-Oya, V., Kim, Y.G., Oh, B.H., and Gruber, S. (2013). An asymmetric SMC-kleisin bridge in prokaryotic condensin. Nat. Struct. Mol. Biol. *20*, 371–379.

Cochran, A.G., and Kim, P.S. (1996). Imitation of Escherichia coli aspartate receptor signaling in engineered dimers of the cytoplasmic domain. Science *271*, 1113–1116.

Cuylen, S., Metz, J., and Haering, C.H. (2011). Condensin structures chromosomal DNA through topological links. Nat. Struct. Mol. Biol. *18*, 894–901.

Decker, B., and Kellermayer, M.S. (2008). Periodically arranged interactions within the myosin filament backbone revealed by mechanical unzipping. J. Mol. Biol. *377*, 307–310.

Delorenzi, M., and Speed, T. (2002). An HMM model for coiled-coil domains and a comparison with PSSM-based predictions. Bioinformatics *18*, 617–625.

Eeftens, J.M., Katan, A.J., Kschonsak, M., Hassler, M., de Wilde, L., Dief, E.M., Haering, C.H., and Dekker, C. (2016). Condensin Smc2-Smc4 dimers are flexible and dynamic. Cell Rep. *14*, 1813–1818.

Elbatsh, A.M., Haarhuis, J.H., Petela, N., Chapard, C., Fish, A., Celie, P.H., Stadnik, M., Ristic, D., Wyman, C., Medema, R.H., et al. (2016). Cohesin releases DNA through asymmetric ATPase-driven ring opening. Mol. Cell *61*, 575–588.

Engler, C., Kandzia, R., and Marillonnet, S. (2008). A one pot, one step, precision cloning method with high throughput capability. PLoS ONE *3*, e3647.

Gligoris, T.G., Scheinost, J.C., Bürmann, F., Petela, N., Chan, K.L., Uluocak, P., Beckouët, F., Gruber, S., Nasmyth, K., and Löwe, J. (2014). Closing the cohesin ring: structure and function of its Smc3-kleisin interface. Science *346*, 963–967.

Goloborodko, A., Imakaev, M.V., Marko, J.F., and Mirny, L. (2016). Compaction and segregation of sister chromatids via active loop extrusion. eLife *5*, 5.

Griese, J.J., Witte, G., and Hopfner, K.P. (2010). Structure and DNA binding activity of the mouse condensin hinge domain highlight common and diverse features of SMC proteins. Nucleic Acids Res. *38*, 3454–3465.

Grigoryan, G., and Degrado, W.F. (2011). Probing designability via a generalized model of helical bundle geometry. J. Mol. Biol. *405*, 1079–1100.

Gruber, S. (2014). Multilayer chromosome organization through DNA bending, bridging and extrusion. Curr. Opin. Microbiol. *22*, 102–110.

Gruber, S., and Errington, J. (2009). Recruitment of condensin to replication origin regions by ParB/SpoOJ promotes chromosome segregation in B. subtilis. Cell *137*, 685–696.

Gruber, M., and Lupas, A.N. (2003). Historical review: another 50th anniversary–new periodicities in coiled coils. Trends Biochem. Sci. *28*, 679–685.

Gruber, S., Haering, C.H., and Nasmyth, K. (2003). Chromosomal cohesin forms a ring. Cell *112*, 765–777.

Gruber, S., Arumugam, P., Katou, Y., Kuglitsch, D., Helmhart, W., Shirahige, K., and Nasmyth, K. (2006). Evidence that loading of cohesin onto chromosomes involves opening of its SMC hinge. Cell *127*, 523–537.

Haering, C.H., and Gruber, S. (2016). SnapShot: SMC protein complexes part I. Cell *164*, 326–326.e1.

Haering, C.H., Löwe, J., Hochwagen, A., and Nasmyth, K. (2002). Molecular architecture of SMC proteins and the yeast cohesin complex. Mol. Cell *9*, 773–788.

Haering, C.H., Schoffnegger, D., Nishino, T., Helmhart, W., Nasmyth, K., and Löwe, J. (2004). Structure and stability of cohesin's Smc1-kleisin interaction. Mol. Cell *15*, 951–964.

Hirano, T. (2016). Condensin-based chromosome organization from bacteria to vertebrates. Cell *164*, 847–857.

Hirano, M., and Hirano, T. (2002). Hinge-mediated dimerization of SMC protein is essential for its dynamic interaction with DNA. EMBO J. *21*, 5733–5744.

Hirano, M., and Hirano, T. (2004). Positive and negative regulation of SMC-DNA interactions by ATP and accessory proteins. EMBO J. *23*, 2664–2673.

Hirano, M., and Hirano, T. (2006). Opening closed arms: long-distance activation of SMC ATPase by hinge-DNA interactions. Mol. Cell *21*, 175–186.

Hirano, M., Anderson, D.E., Erickson, H.P., and Hirano, T. (2001). Bimodal activation of SMC ATPase by intra- and inter-molecular interactions. EMBO J. *20*, 3238–3250.

Hitchcock-DeGregori, S.E., and Varnell, T.A. (1990). Tropomyosin has discrete actin-binding sites with sevenfold and fourteenfold periodicities. J. Mol. Biol. *214*, 885–896.

Hohl, M., Kwon, Y., Galván, S.M., Xue, X., Tous, C., Aguilera, A., Sung, P., and Petrini, J.H. (2011). The Rad50 coiled-coil domain is indispensable for Mre11 complex functions. Nat. Struct. Mol. Biol. *18*, 1124–1131.

Hohl, M., Kochańczyk, T., Tous, C., Aguilera, A., Krężel, A., and Petrini, J.H. (2015). Interdependence of the rad50 hook and globular domain functions. Mol. Cell *57*, 479–491.

Hopfner, K.P., Craig, L., Moncalian, G., Zinkel, R.A., Usui, T., Owen, B.A., Karcher, A., Henderson, B., Bodmer, J.L., McMurray, C.T., et al. (2002). The Rad50 zinc-hook is a structure joining Mre11 complexes in DNA recombination and repair. Nature *418*, 562–566.

Hu, B., Itoh, T., Mishra, A., Katoh, Y., Chan, K.L., Upcher, W., Godlee, C., Roig, M.B., Shirahige, K., and Nasmyth, K. (2011). ATP hydrolysis is required for relocating cohesin from sites occupied by its Scc2/4 loading complex. Curr. Biol. *21*, 12–24.

Huber, R.G., Kulemzina, I., Ang, K., Chavda, A.P., Suranthran, S., Teh, J.T., Kenanov, D., Liu, G., Rancati, G., Szmyd, R., et al. (2016). Impairing cohesin Smc1/3 head engagement compensates for the lack of Eco1 function. Structure *24*, 1991–1999.

Huis in 't Veld, P.J., Herzog, F., Ladurner, R., Davidson, I.F., Piric, S., Kreidl, E., Bhaskara, V., Aebersold, R., and Peters, J.M. (2014). Characterization of a DNA exit gate in the human cohesin ring. Science *346*, 968–972.

Ivanov, D., and Nasmyth, K. (2005). A topological interaction between cohesin rings and a circular minichromosome. Cell *122*, 849–860.

Jeppsson, K., Kanno, T., Shirahige, K., and Sjögren, C. (2014). The maintenance of chromosome structure: positioning and functioning of SMC complexes. Nat. Rev. Mol. Cell Biol. *15*, 601–614.

Kamada, K., Miyata, M., and Hirano, T. (2013). Molecular basis of SMC ATPase activation: role of internal structural changes of the regulatory subcomplex ScpAB. Structure *21*, 581–594.

Kornberg, A., and Pricer, W.E., Jr. (1951). Enzymatic phosphorylation of adenosine and 2,6-diaminopurine riboside. J. Biol. Chem. *193*, 481–495.

Lammens, A., Schele, A., and Hopfner, K.P. (2004). Structural biochemistry of ATP-driven dimerization and DNA-stimulated activation of SMC ATPases. Curr. Biol. *14*, 1778–1782.

Langmead, B., and Salzberg, S.L. (2012). Fast gapped-read alignment with Bowtie 2. Nat. Methods *9*, 357–359.

Le, T.B., Imakaev, M.V., Mirny, L.A., and Laub, M.T. (2013). High-resolution mapping of the spatial organization of a bacterial chromosome. Science *342*, 731–734.

Liu, Y., and Schmidt, B. (2014). Multiple protein sequence alignment with MSAProbs. Methods Mol. Biol. *1079*, 211–218.

Lupas, A.N., and Gruber, M. (2005). The structure of α-helical coiled coils. Adv. Protein Chem. *70*, 37–78.

Mannini, L., Cucco, F., Quarantotti, V., Krantz, I.D., and Musio, A. (2013). Mutation spectrum and genotype-phenotype correlation in Cornelia de Lange syndrome. Hum. Mutat. *34*, 1589–1596.

Marbouty, M., Le Gall, A., Cattoni, D.I., Cournac, A., Koh, A., Fiche, J.B., Mozziconacci, J., Murray, H., Koszul, R., and Nollmann, M. (2015). Condensin- and replication-mediated bacterial chromosome folding and origin condensation revealed by Hi-C and super-resolution imaging. Mol. Cell *59*, 588–602.

Mascarenhas, J., Soppa, J., Strunnikov, A.V., and Graumann, P.L. (2002). Cell cycle-dependent localization of two novel prokaryotic chromosome segregation and condensation proteins in Bacillus subtilis that interact with SMC protein. EMBO J. *21*, 3108–3118.

Mascarenhas, J., Volkov, A.V., Rinn, C., Schiener, J., Guckenberger, R., and Graumann, P.L. (2005). Dynamic assembly, localization and proteolysis of the Bacillus subtilis SMC complex. BMC Cell Biol. *6*, 28.

McLachlan, A.D., and Karn, J. (1982). Periodic charge distributions in the myosin rod amino acid sequence match cross-bridge spacings in muscle. Nature *299*, 226–231.

Merkenschlager, M., and Nora, E.P. (2016). CTCF and cohesin in genome folding and transcriptional gene regulation. Annu. Rev. Genomics Hum. Genet. *17*, 17–43.

Minnen, A., Attaiech, L., Thon, M., Gruber, S., and Veening, J.W. (2011). SMC is recruited to oriC by ParB and promotes chromosome segregation in Streptococcus pneumoniae. Mol. Microbiol. *81*, 676–688.

Minnen, A., Bürmann, F., Wilhelm, L., Anchimiuk, A., Diebold-Durand, M.L., and Gruber, S. (2016). Control of Smc coiled coil architecture by the ATPase heads facilitates targeting to chromosomal ParB/parS and release onto flanking DNA. Cell Rep. *14*, 2003–2016.

Möglich, A., Ayers, R.A., and Moffat, K. (2009). Design and signaling mechanism of light-regulated histidine kinases. J. Mol. Biol. *385*, 1433–1444.

Murayama, Y., and Uhlmann, F. (2015). DNA entry into and exit out of the cohesin ring by an interlocking gate mechanism. Cell *163*, 1628–1640.

Nasmyth, K. (2001). Disseminating the genome: joining, resolving, and separating sister chromatids during mitosis and meiosis. Annu. Rev. Genet. *35*, 673–745.

Nolivos, S., and Sherratt, D. (2014). The bacterial chromosome: architecture and action of bacterial SMC and SMC-like complexes. FEMS Microbiol. Rev. *38*, 380–392.

Orgil, O., Mor, H., Matityahu, A., and Onn, I. (2016). Identification of a region in the coiled-coil domain of Smc3 that is essential for cohesin activity. Nucleic Acids Res. *44*, 6309–6317.

Palecek, J.J., and Gruber, S. (2015). Kite proteins: a superfamily of SMC/kleisin partners conserved across bacteria, archaea, and eukaryotes. Structure *23*, 2183–2190.

Peters, J.M., and Nishiyama, T. (2012). Sister chromatid cohesion. Cold Spring Harb. Perspect. Biol. *4*, 4.

Pu, W.T., and Struhl, K. (1991). The leucine zipper symmetrically positions the adjacent basic regions for specific DNA binding. Proc. Natl. Acad. Sci. U S A *88*, 6901–6905.

Schwartz, M.A., and Shapiro, L. (2011). An SMC ATPase mutant disrupts chromosome segregation in Caulobacter. Mol. Microbiol. *82*, 1359–1374.

Sievers, F., Wilm, A., Dineen, D., Gibson, T.J., Karplus, K., Li, W., Lopez, R., McWilliam, H., Remmert, M., Söding, J., et al. (2011). Fast, scalable generation of high-quality protein multiple sequence alignments using Clustal Omega. Mol. Syst. Biol. *7*, 539.

Söding, J., Remmert, M., Biegert, A., and Lupas, A.N. (2006). HHsenser: exhaustive transitive profile search using HMM-HMM comparison. Nucleic Acids Res. *34*, W374–W378.

Soh, Y.M., Bürmann, F., Shin, H.C., Oda, T., Jin, K.S., Toseland, C.P., Kim, C., Lee, H., Kim, S.J., Kong, M.S., et al. (2015). Molecular basis for SMC rod formation and its dissolution upon DNA binding. Mol. Cell *57*, 290–303.

Soppa, J., Kobayashi, K., Noirot-Gros, M.F., Oesterhelt, D., Ehrlich, S.D., Dervyn, E., Ogasawara, N., and Moriya, S. (2002). Discovery of two novel families of proteins that are proposed to interact with prokaryotic SMC proteins, and characterization of the Bacillus subtilis family members ScpA and ScpB. Mol. Microbiol. *45*, 59–71.

Spiess, A.N., Feig, C., and Ritz, C. (2008). Highly accurate sigmoidal fitting of real-time PCR data by introducing a parameter for asymmetry. BMC Bioinformatics *9*, 221.

Stigler, J., Çamdere, G.O., Koshland, D.E., and Greene, E.C. (2016). Single-molecule imaging reveals a collapsed conformational state for DNA-bound cohesin. Cell Rep. *15*, 988–998.

Sullivan, N.L., Marquis, K.A., and Rudner, D.Z. (2009). Recruitment of SMC by ParB-parS organizes the origin region and promotes efficient chromosome segregation. Cell *137*, 697–707.

Truebestein, L., and Leonard, T.A. (2016). Coiled-coils: the long and short of it. BioEssays *38*, 903–916.

Umbarger, M.A., Toro, E., Wright, M.A., Porreca, G.J., Baù, D., Hong, S.H., Fero, M.J., Zhu, L.J., Marti-Renom, M.A., McAdams, H.H., et al. (2011). The three-dimensional architecture of a bacterial genome and its alteration by genetic perturbation. Mol. Cell *44*, 252–264.

Waldman, V.M., Stanage, T.H., Mims, A., Norden, I.S., and Oakley, M.G. (2015). Structural mapping of the coiled-coil domain of a bacterial condensin and comparative analyses across all domains of life suggest conserved features of SMC proteins. Proteins *83*, 1027–1045.

Wang, X., Le, T.B., Lajoie, B.R., Dekker, J., Laub, M.T., and Rudner, D.Z. (2015). Condensin promotes the juxtaposition of DNA flanking its loading site in Bacillus subtilis. Genes Dev. *29*, 1661–1675.

Wells, J.N., Gligoris, T.G., Nasmyth, K.A., and Marsh, J.A. (2017). Evolution of condensin and cohesin complexes driven by replacement of Kite by Hawk proteins. Curr. Biol. *27*, R17–R18.

White, G.E., and Erickson, H.P. (2006). Sequence divergence of coiled coils–structural rods, myosin filament packing, and the extraordinary conservation of cohesins. J. Struct. Biol. *154*, 111–121.

White, G.E., and Erickson, H.P. (2009). The coiled coils of cohesin are conserved in animals, but not in yeast. PLoS ONE *4*, e4674.

Wilhelm, L., Bürmann, F., Minnen, A., Shin, H.C., Toseland, C.P., Oh, B.H., and Gruber, S. (2015). SMC condensin entraps chromosomal DNA by an ATP hydrolysis dependent loading mechanism in Bacillus subtilis. eLife *4*, 4.

Wolgemuth, C.W., and Sun, S.X. (2006). Elasticity of α-helical coiled coils. Phys. Rev. Lett. *97*, 248101.

Woo, J.S., Lim, J.H., Shin, H.C., Suh, M.K., Ku, B., Lee, K.H., Joo, K., Robinson, H., Lee, J., Park, S.Y., et al. (2009). Structural studies of a bacterial condensin complex reveal ATP-dependent disruption of intersubunit interactions. Cell *136*, 85–96.

STAR★METHODS

KEY RESOURCES TABLE

REAGENT or RESOURCE	SOURCE	IDENTIFIER
Antibodies		
Anti-ScpB-His6 rabbit antiserum	The Gruber Laboratory	COD003
Anti-Smc polyclonal rabbit antibody, affinity purified	The Gruber Laboratory	COD006
Chemicals, Peptides, and Recombinant Proteins		
Adenosine triphosphate (ATP)	Sigma-Aldrich	Cat#A6419-10G
Bis(maleimido)ethane (BMOE)	Thermo Scientific	Cat#22323
*Bsa*I	New England Biolabs	Cat#R0535L
*Bsg*I	New England Biolabs	Cat#R0559L
Certified Low Melt Agarose	Bio-Rad Laboratories	Cat#161-3111
Dynabeads Protein-G	Life Technologies	Cat#10004D
Erythromycin	AppliChem	Cat#A2275,0005
GlycoBlue	Ambion	Cat#AM9515
HaloTag Oregon Green Ligand	Promega	Cat#G2802
HaloTag TMR Ligand	Promega	Cat#G8251
HiTrap Blue HP	GE Healthcare	Cat#17-0413-01
HiTrap Heparin HP	GE Healthcare	Cat#17-0407-01
Lincomycin	AppliChem	Cat#A7697,0005
Nicotinamide adenine dinucleotide (NADH)	Sigma-Aldrich	Cat#N8129-100MG
Overnight Express Instant TB Medium	Merck	Cat#71491-5
Oxoid Nutrient Agar (ONA)	Oxoid	Cat#CM003
Phosphoenolpyruvic acid (PEP)	Sigma-Aldrich	Cat#P7002-100MG
Phusion HotStart II DNA Polymerase	Thermo Scientific	Cat#F-549L
Protease Inhibitor Cocktail	Sigma-Aldrich	Cat#P8849-5ML
Pyruvate kinase/lactate dehydrogenase	Sigma-Aldrich	Cat#P0294-5ML
Ready-Lyse Lysozyme Solution	Epicenter	Cat#R1802M
Sm DNase	MPIB Core Facility	SmDNase
Superose 6 Prep Grade	GE Healthcare	Cat#17-0489-01
T4 DNA Ligase	Thermo Scientific	Cat#EL0016
Taq DNA Polymerase	New England Biolabs	Cat#M0267S
Critical Commercial Assays		
No ROX SYBR MasterMix blue dTTP	Takyon	Cat#UF-NSMT-B0701
NucleoFast 96 PCR Plate	Macherey-Nagel	Cat#743100.1
EZ-Tn5 < KAN-2 > Insertion Kit	Epicenter	Cat#EZI982K
NuPAGE 3-8% Tris-Acetate Gels	Life Technologies	Cat#EA03755BOX
QIAquick PCR Purification Kit	QIAGEN	Cat#28106
Costar Spin-X Centrifuge Tube Filter	Corning	Cat#8163
Ovation Ultralow System V2	NuGEN	Cat#0344
NEXTflex PCR-Free DNA Sequencing Kit	Bioo Scientific	Cat#5142-01
Deposited Data		
ChIP-seq data	This paper	SRA: SRP094054
Insertion library sequencing data	This paper	SRA: SRP094088
Bacillus subtilis reference genome	NCBI	NC_000964
Experimental Models: Organisms/Strains		
E. coli: BL21-Gold (DE3)	MPIB Core Facility	N/A

(*Continued on next page*)

Continued

REAGENT or RESOURCE	SOURCE	IDENTIFIER
B. subtilis: 1A700, smc ftsY::ermB, trpC2	The Gruber Laboratory	BSG1002
B. subtilis: 1A700, Δsmc ftsY::ermB, trpC2	The Gruber Laboratory	BSG1007
B. subtilis: 1A700, smc(E1118Q) ftsY::ermB, trpC2	The Gruber Laboratory	BSG1008
B. subtilis: 1A700, smc(S1090R) ftsY::ermB, trpC2	The Gruber Laboratory	BSG1046
B. subtilis: 1A700, smc(Pf Rad50 Zinc hook) ftsY::ermB, trpC2	This paper	BSG1075
B. subtilis: 1A700, smc(C119S, C437S, C826S, C1114S)-TEV-His12-HaloTag(C61V, C262A) ftsY::ermB, trpC2	The Gruber Laboratory	BSG1360
B. subtilis: 1A700, smc(C119S, C437S, C826S, C1114S, K1151C)-TEV-His12-HaloTag(C61V, C262A) ftsY::ermB, trpC2	The Gruber Laboratory	BSG1457
B. subtilis: 1A700, smc(C119S, C437S, C826S, C1114S, K1151C, E1118Q)-TEV-His12-HaloTag(C61V, C262A) ftsY::ermB, trpC2	The Gruber Laboratory	BSG1488
B. subtilis: 1A700, smc(C119S, C437S, G657A, G658A, G662A, G663A, C826S, C1114S, E1118Q, K1151C)-TEV-His12-HaloTag(C61V, C262A) ftsY::ermB, trpC2	The Gruber Laboratory	BSG1598
B. subtilis: 1A700, smc(C119S, C437S, C826S, S1090R, C1114S, K1151C)-TEV-His12-HaloTag(C61V, C262A) ftsY::ermB, trpC2	The Gruber Laboratory	BSG1600
B. subtilis: 1A700, smc(C119S, C437S, R558C, N634C, C826S, C1114S)-TEV-His12-HaloTag(C61V, C262A) ftsY::ermB, trpC2	The Gruber Laboratory	BSG1638
B. subtilis: 1A700, smc(S19C, R558C, N634C, R1032C)-TEV-HaloTag ftsY::ermB, cat::scpA(E52C, H235C), dnaN(N114C, V313C)::specR, trpC2	The Gruber Laboratory	BSG1782
B. subtilis: 1A700, smc(S19C, K37I, R558C, N634C, R1032C)-TEV-HaloTag ftsY::ermB, cat::scpA(E52C, H235C), dnaN(N114C, V313C)::specR, trpC2	The Gruber Laboratory	BSG1784
B. subtilis: 1A700, smc(S19C, R558C, N634C, R1032C, E1118Q)-TEV-HaloTag ftsY::ermB, cat::scpA(E52C, H235C), dnaN(N114C, V313C)::specR, trpC2	The Gruber Laboratory	BSG1786
B. subtilis: 1A700, smc(494-GGSGGSGGSGG, 678-GGSGGSGGSGG) ftsY::ermB, trpC2	This paper	BSG1835
B. subtilis: 1A700, smc ftsY::ermB, specR::scpA ΔscpB, trpC2	The Gruber Laboratory	BSG1891
B. subtilis: 1A700, Δsmc ftsY::specR, trpC2	This paper	BSG1919
B. subtilis: 1A700, smc(C119S, C437S, A715C, C826S, C1114S)-TEV-His12-HaloTag(C61V, C262A) ftsY::ermB, trpC2	The Gruber Laboratory	BSG1921
B. subtilis: 1A700, rncS smc(Δhinge) ftsY::tetL, trpC2	This paper	BSG1957
B. subtilis: 1A700, smc(1-392)-SGPGGGGGRQNSQ-smc(393-1186) ftsY::ermB, trpC2	This paper	BSG2017
B. subtilis: 1A700, smc(1-394)-SGPGGGGGRQQAS-smc(395-1186) ftsY::ermB, trpC2	This paper	BSG2018
B. subtilis: 1A700, smc(1-479)-SGPGGGGGRQYQA-smc(480-1186) ftsY::ermB, trpC2	This paper	BSG2021
B. subtilis: 1A700, smc(1-725)-SGPGGGGGRQGLR-smc(726-1186) ftsY::ermB, trpC2	This paper	BSG2026
B. subtilis: 1A700, smc(1-480, 487-684, 690-1186) ftsY::ermB, trpC2	This paper	BSG2088
B. subtilis: 1A700, smc(1-463, 487-684, 708-1186) ftsY::ermB, trpC2	This paper	BSG2089
B. subtilis: 1A700, smc(1-438, 487-684, 733-1186) ftsY::ermB, trpC2	This paper	BSG2090
B. subtilis: 1A700, smc(1-435, 487-684, 736-1186) ftsY::ermB, trpC2	This paper	BSG2091
B. subtilis: 1A700, smc(1-399, 487-684, 772-1186) ftsY::ermB, trpC2	This paper	BSG2092
B. subtilis: 1A700, smc(1-395, 487-684, 776-1186) ftsY::ermB, trpC2	This paper	BSG2093
B. subtilis: 1A700, smc(1-359, 487-684, 815-1186) ftsY::ermB, trpC2	This paper	BSG2094
B. subtilis: 1A700, smc(1-356, 487-684, 818-1186) ftsY::ermB, trpC2	This paper	BSG2104
B. subtilis: 1A700, smc(1-480, 487-684, 690-1186, C119S, C437S, R558C, N634C, C826S, C1114S)-TEV-His12-HaloTag(C61V, C262A) ftsY::ermB, trpC2	This paper	BSG2118

(*Continued on next page*)

Continued

REAGENT or RESOURCE	SOURCE	IDENTIFIER
B. subtilis: 1A700, smc(1-463, 487-684, 708-1186, C119S, C437S, R558C, N634C, C826S, C1114S)-TEV-His12-HaloTag(C61V, C262A) ftsY::ermB, trpC2	This paper	BSG2119
B. subtilis: 1A700, smc(1-435, 487-684, 736-1186, C119S, R558C, N634C, C826S, C1114S)-TEV-His12-HaloTag(C61V, C262A) ftsY::ermB, trpC2	This paper	BSG2120
B. subtilis: 1A700, smc(1-399, 487-684, 772-1186, C119S, R558C, N634C, C826S, C1114S)-TEV-His11-HaloTag(C61V, C262A) ftsY::ermB, trpC2	This paper	BSG2121
B. subtilis: 1A700, smc(1-395, 487-684, 776-1186, C119S, R558C, N634C, C826S, C1114S)-TEV-His12-HaloTag(C61V, C262A) ftsY::ermB, trpC2	This paper	BSG2122
B. subtilis: 1A700, smc(1-480, 487-684, 690-1186, C119S, C437S, C826S, C1114S, E1118Q, K1151C)-TEV-His12-HaloTag(C61V, C262A) ftsY::ermB, trpC2	This paper	BSG2133
B. subtilis: 1A700, smc(1-463, 487-684, 708-1186, C119S, C437S, C826S, C1114S, E1118Q, K1151C)-TEV-His12-HaloTag(C61V, C262A) ftsY::ermB, trpC2	This paper	BSG2134
B. subtilis: 1A700, smc(1-435, 487-684, 736-1186, C119S, C826S, C1114S, E1118Q, K1151C)-TEV-His12-HaloTag(C61V, C262A) ftsY::ermB, trpC2	This paper	BSG2135
B. subtilis: 1A700, smc(1-399, 487-684, 772-1186, C119S, C826S, C1114S, E1118Q, K1151C)-TEV-His12-HaloTag(C61V, C262A) ftsY::ermB, trpC2	This paper	BSG2136
B. subtilis: 1A700, smc(1-395, 487-684, 776-1186, C119S, C826S, C1114S, E1118Q, K1151C)-TEV-His12-HaloTag(C61V, C262A) ftsY::ermB, trpC2	This paper	BSG2137
B. subtilis: 1A700, smc(1-486, SpnSmc(398-768), 685-1186) ftsY::ermB, trpC2	This paper	BSG2348
B. subtilis: 1A700, smc(1-483, SpnSmc(398-768), 688-1186) ftsY::ermB, trpC2	This paper	BSG2349
B. subtilis: 1A700, smc(1-435, SpnSmc(398-768), 736-1186) ftsY::ermB, trpC2	This paper	BSG2350
B. subtilis: 1A700, smc(1-399, SpnSmc(398-768), 772-1186) ftsY::ermB, trpC2	This paper	BSG2351
B. subtilis: 1A700, smc(1-395, SpnSmc(398-768), 776-1186) ftsY::ermB, trpC2	This paper	BSG2352
B. subtilis: 1A700, smc(1-349, SpnSmc(398-768), 825-1186) ftsY::ermB, trpC2	This paper	BSG2353
B. subtilis: 1A700, smc(1-321, SpnSmc(398-768), 853-1186) ftsY::ermB, trpC2	This paper	BSG2354
B. subtilis: 1A700, smc(1-438, SpnSmc(398-768), 733-1186) ftsY::ermB, trpC2	This paper	BSG2355
B. subtilis: 1A700, smc(1-347, SpnSmc(398-768), 829-1186) ftsY::ermB, trpC2	This paper	BSG2356
B. subtilis: 1A700, smc(1-438, 487-684, 733-1186, C119S, C437S, R558C, N634C, C826S, C1114S)-TEV-His12-HaloTag(C61V, C262A) ftsY::ermB, trpC2	This paper	BSG2403
B. subtilis: 1A700, smc(1-435, 487-684, 736-1186, C119S, C826S, C1114S, K1151C)-TEV-His12-HaloTag(C61V, C262A) ftsY::ermB, trpC2	This paper	BSG2408
B. subtilis: 1A700, smc(1-438, 487-684, 733-1186, S1090R) ftsY::ermB, trpC2	This paper	BSG2409
B. subtilis: 1A700, smc(1-435, 487-684, 736-1186, S1090R) ftsY::ermB, trpC2	This paper	BSG2410
B. subtilis: 1A700, smc(1-480, Pf Rad50 Zinc hook, 691-1186) ftsY::ermB, trpC2	This paper	BSG2414
B. subtilis: 1A700, smc(1-463, Pf Rad50 Zinc hook, 708-1186) ftsY::ermB, trpC2	This paper	BSG2415
B. subtilis: 1A700, smc(1-438, Pf Rad50 Zinc hook, 733-1186) ftsY::ermB, trpC2	This paper	BSG2416
B. subtilis: 1A700, smc(1-435, Pf Rad50 Zinc hook, 736-1186) ftsY::ermB, trpC2	This paper	BSG2417
B. subtilis: 1A700, smc(1-427, Pf Rad50 Zinc hook, 744-1186) ftsY::ermB, trpC2	This paper	BSG2418
B. subtilis: 1A700, smc(1-398, Pf Rad50 Zinc hook, 773-1186) ftsY::ermB, trpC2	This paper	BSG2419
B. subtilis: 1A700, smc(1-462, 487-684, 709-1186) ftsY::ermB, trpC2	This paper	BSG2479
B. subtilis: 1A700, smc(1-462, 487-684, 709-1186, L525H) ftsY::ermB, trpC2	This paper	BSG2480
B. subtilis: 1A700, smc(1-462, 487-684, 709-1186, Q547R) ftsY::ermB, trpC2	This paper	BSG2481
B. subtilis: 1A700, smc(1-458, 487-684, 713-1186) ftsY::ermB, trpC2	This paper	BSG2482
B. subtilis: 1A700, smc(1-458, 487-684, 713-1186, L525H) ftsY::ermB, trpC2	This paper	BSG2483
B. subtilis: 1A700, smc(1-458, 487-684, 713-1186, Q547R) ftsY::ermB, trpC2	This paper	BSG2484

(*Continued on next page*)

Continued

REAGENT or RESOURCE	SOURCE	IDENTIFIER
B. subtilis: 1A700, smc(C119S, C437S, T495C, C826S, C1114S)-TEV-His12-HaloTag(C61V, C262A) ftsY::ermB, trpC2	This paper	BSG2485
B. subtilis: 1A700, smc(1-462, 487-684, 709-1186, C119S, C437S, T495C, C826S, C1114S)-TEV-His12-HaloTag(C61V, C262A) ftsY::ermB, trpC2	This paper	BSG2486
B. subtilis: 1A700, smc(1-462, 487-684, 709-1186, C119S, C437S, T495C, L525H, C826S, C1114S)-TEV-His12-HaloTag(C61V, C262A) ftsY::ermB, trpC2	This paper	BSG2487
B. subtilis: 1A700, smc(1-462, 487-684, 709-1186, C119S, C437S, T495C, Q547R, C826S, C1114S)-TEV-His12-HaloTag(C61V, C262A) ftsY::ermB, trpC2	This paper	BSG2488
B. subtilis: 1A700, smc(1-458, 487-684, 713-1186, C119S, C437S, T495C, C826S, C1114S)-TEV-His12-HaloTag(C61V, C262A) ftsY::ermB, trpC2	This paper	BSG2492
B. subtilis: 1A700, smc(1-458, 487-684, 713-1186, C119S, C437S, T495C, L525H, C826S, C1114S)-TEV-His12-HaloTag(C61V, C262A) ftsY::ermB, trpC2	This paper	BSG2493
B. subtilis: 1A700, smc(1-458, 487-684, 713-1186, C119S, C437S, T495C, Q547R, C826S, C1114S)-TEV-His12-HaloTag(C61V, C262A) ftsY::ermB, trpC2	This paper	BSG2494
B. subtilis: 1A700, smc(1-438, 487-684, 733-1186, C119S, C437S, C826S, C1114S, K1151C)-TEV-His12-HaloTag(C61V, C262A) ftsY::ermB, trpC2	This paper	BSG2511
B. subtilis: 1A700, smc(Pf Rad50 Zinc hook, C119S, C437S, C826S, C1114S)-TEV-His12-HaloTag(C61V, C262A) ftsY::ermB, trpC2	This paper	BSG2512
B. subtilis: 1A700, smc(Pf Rad50 Zinc hook, C119S, C437S, A715C, C826S, C1114S)-TEV-His12-HaloTag(C61V, C262A) ftsY::ermB, trpC2	This paper	BSG2513
B. subtilis: 1A700, smc(1-438, 487-684, 733-1186, C119S, T495C, C826S, C1114S)-TEV-His12-HaloTag(C61V, C262A) ftsY::ermB, trpC2	This paper	BSG2531
B. subtilis: 1A700, smc(1-462, 487-684, 709-1186, D280G) ftsY::ermB, trpC2	This paper	BSG2578
B. subtilis: 1A700, smc(1-462, 487-684, 709-1186, Q320R) ftsY::ermB, trpC2	This paper	BSG2579
B. subtilis: 1A700, smc(1-462, 487-684, 709-1186, E323K) ftsY::ermB, trpC2	This paper	BSG2580
B. subtilis: 1A700, smc(1-438, 487-684, 733-1186, S19C, R558C, N634C, R1032C)-TEV-HaloTag ftsY::ermB, cat::scpA(E52C, H235C), dnaN(N114C, V313C)::specR, trpC2	This paper	BSG2617
B. subtilis: 1A700, smc(1-435, 487-684, 736-1186, S19C, R558C, N634C, R1032C)-TEV-HaloTag ftsY::ermB, cat::scpA(E52C, H235C), dnaN(N114C, V313C)::specR, trpC2	This paper	BSG2618
B. subtilis: 1A700, smc(1-399, 487-684, 772-1186, S19C, R558C, N634C, R1032C)-TEV-HaloTag ftsY::ermB, cat::scpA(E52C, H235C), dnaN(N114C, V313C)::specR, trpC2	This paper	BSG2619
B. subtilis: 1A700, smc(1-395, 487-684, 776-1186, S19C, R558C, N634C, R1032C)-TEV-HaloTag ftsY::ermB, cat::scpA(E52C, H235C), dnaN(N114C, V313C)::specR, trpC2	This paper	BSG2620
Recombinant DNA		
pSG682 pJET1.2 ermB cassette	This paper	pSG682
pSG841 pJET1.2 ylqB region	This paper	pSG841
pSG849 pJET1.2 ftsY region	This paper	pSG849
pSG956 pJET1.2 PfRad50 zinc hook	This paper	pSG956
pSG1134 pUC19 ‘rncS smc locus with ermB	This paper	pSG1134
pSG1497 pET-22b Smc	This paper	pSG1497
pSG1525 pET-Gate2 mazEF	This paper	pSG1525
pSG1580 pJET1.2 BsSmc hinge	This paper	pSG1580
pSG2356 pJET1.2 (398-768)SpSmc hinge-coils	This paper	pSG2356
pSG2914 pET-Gold1 Smc(1-438, 487-684, 733-1186)	This paper	pSG2914
pSG2915 pET-Gold1 Smc(1-435, 487-684, 736-1186)	This paper	pSG2915
pSG2916 pET-Gold1 Smc(1-399, 487-684, 772-1186)	This paper	pSG2916
pSG2917 pET-Gold1 Smc(1-395, 487-684, 776-1186)	This paper	pSG2917

(*Continued on next page*)

Continued		
REAGENT or RESOURCE	SOURCE	IDENTIFIER
pSG2920 pET-Gold1 Smc(K37I, 1-463, 487-684, 708-1186)	This paper	pSG2920
pSG2921 pET-Gold1 Smc(K37I, 1-438, 487-684, 733-1186)	This paper	pSG2921
pSG2965 pET-Gold1 Smc(394-SGPGGGGGRQ)	This paper	pSG2965
pSG2966 pET-Gold1 Smc(479-SGPGGGGGRQ)	This paper	pSG2965
Oligonucleotides		
qPCR primers, see Table S2	This paper	N/A
PCR primers for HTP genetic engineering, see Table S5	This paper	N/A
Software and Algorithms		
Bowtie2 v2.2.5	Langmead and Salzberg, 2012	http://bowtie-bio.sourceforge.net/bowtie2/index.shtml
BLAST v2.3.0	NCBI	ftp://ftp.ncbi.nlm.nih.gov/blast/executables/blast+/
Clustal Omega v1.2.0	Sievers et al., 2011	http://www.clustal.org/omega/
HHSenser webserver	Söding et al., 2006	https://toolkit.tuebingen.mpg.de/hhsenser
MSAProbs v0.9.7	Liu and Schmidt, 2014	http://msaprobs.sourceforge.net/homepage.htm
Wolfram Mathematica	Wolfram Research Inc.	http://www.wolfram.com/mathematica/
Wolfram Language package for the analysis of insertion screens	This paper	https://github.com/fbuermann/InsertionMapping
Other		
Coiled-coil length prediction data, see Table S4	This paper	N/A
HTP genetic engineering data, see Table S5	This paper	N/A
Insertion screen data, see Table S6	This paper	N/A

CONTACT FOR REAGENT AND RESOURCE SHARING

Further information and requests for reagents may be directed to, and will be fulfilled by the Lead Contact, Stephan Gruber (stephan.gruber@unil.ch).

EXPERIMENTAL MODEL AND SUBJECT DETAILS

Bacillus subtilis Strains and Growth

B. subtilis strains are based on the parental strain 1A700. Allelic replacement was performed by double-crossover recombination at the endogenous *smc* locus using natural competence (Bürmann et al., 2013). Transformants were selected on SMG solid medium with appropriate antibiotics. Strains were single-colony purified and verified by a combination of marker testing, phenotype testing, PCR and Sanger sequencing where appropriate. For dilution spot assays cells were grown to stationary phase in liquid SMG and 9^2 and 9^5 fold dilutions were spotted onto solid medium (Bürmann et al., 2013). Strain usage for all reported experiments is listed in Table S1.

METHOD DETAILS

Protein Sequence Analysis

The super-helical period of the Smc coiled coil was estimated with CCCP (Grigoryan and Degrado, 2011) using the coiled coil from PDB: 4RSJ. Coiled-coil probabilities were computed with Marcoil (Delorenzi and Speed, 2002).

Sets of diverged SMC and SMC-like sequences were obtained as follows (Figure S1A). First, a reference multiple sequence alignment (MSA) of 18 SMC hinges (6 bacterial, 6 archaeal, 6 eukaryotic) was constructed with MSA-Probs (Liu and Schmidt, 2014). Reference alignments for MukB, MksB and Rad50 were similarly constructed using sequences of the respective dimerization domains. Then, the alignments were used as queries for HHSenser searches (Söding et al., 2006). The resulting sequence sets were filtered with PSI-BLAST for members containing significant homology to reference MSAs of N-terminal head (HeadN) and C-terminal head (HeadC) with a threshold of $E < 1$. For Rad50 proteins, sequences were discarded that displayed $E < 1$ with the SMC hinge reference MSA.

For coiled-coil length estimation, batches of 200 sequences were aligned to reference MSAs for HeadN, HeadC and dimerization domain using Clustal Omega (Sievers et al., 2011). Sequences were additionally filtered as follows: In the domain reference MSAs, all positions were chosen that contained non-gap residues in at least 75% of the reference sequences. Then, target sequences were picked from the Clustal MSAs that had non-gap residues in at least 75% of those positions. Domain boundaries were defined as the outermost residues aligning to the reference MSAs. N- and C-terminal coiled-coil strands were defined as the interlinking regions between the head regions and the dimerization domain. Sequences were discarded for which the length of the shorter coiled-coil strand was less than 75% of the length of the longer strand. Arm length was defined as the mean length of N- and C-terminal coiled-coil strands.

Finally, sequence sets were filtered by classification as described below. Prokaryotic SMC, MukB and MksB were classified as either Smc, MukB or MksB. For each of those sets, retrieved sequences belonging to non-target classes were discarded. Eukaryotic SMC were either classified as Smc1, Smc2, Smc3, Smc4, Smc5, or Smc6. Classification of Rad50 proteins was omitted. Sequence classification was performed as follows: For each protein class, sequences for HeadN, Hinge and HeadC were extracted from four reference sequences. For each unknown protein, the corresponding domain sequences were extracted and Smith-Waterman similarities to the reference domains were computed using the BLOSUM62 matrix. Similarity scores were normalized for domain length, and the class that obtained the highest average similarity score was defined as the protein class. Datasets are listed in Table S4.

High-throughput Allelic Replacement Screening

PCR primers were designed based on disulphide mapping of the Smc coiled-coil register (Minnen et al., 2016), and PCRs for 5′- and 3′-regions of the *smc* gene were performed in 96-well plates using Phusion DNA Polymerase (New England Biolabs). DNA was purified in NucleoFast 96 PCR plates (Macherey-Nagel). Circular targeting constructs were assembled in Golden Gate reactions using *Bsa*I and T4 DNA ligase (Engler et al., 2008) with cloned and sequence verified modules for the dimerization domain (pSG956, pSG1580, pSG2356), the downstream *ftsY* gene (pSG849), an *ermB* marker cassette (pSG682), a downstream homology region (pSG841), and a non-replicating plasmid backbone containing a *mazF* toxin gene (pSG1525) (Figure S2A). The *mazF* gene was used to efficiently counter-select single-crossover integration. Reaction mixtures were transformed into either a *smc* deletion strain (BSG1919; for the truncation screen with a wild-type Smc hinge) or a *smc* null strain lacking the hinge region of the *smc* gene (BSG1957; for all other high-throughput assays). The latter approach was chosen due to larger homology for double-crossover recombination resulting in improved transformation efficiencies. Note that the hinge-deletion strain cannot regenerate a wild-type allele from the transformed constructs (unless the construct encodes wild-type Smc) due to missing homology. Transformants were selected on Oxoid nutrient agar (ONA) with 0.4 μg/mL erythromycin and 10 μg/mL lincomycin at 37°C.

Plates were imaged 36 hr after transformation. Colonies were identified and quantified by an automated segmentation approach in Wolfram Mathematica. Briefly, the position of the plate was determined in the images, positions of small ellipsoid objects on the plate were identified, and objects were classified into colony and non-colony groups using the built-in Classify function and a small training set. The total area of colonies per plate was obtained and was scaled to metric dimensions by using the known diameter of the plate. Datasets are listed in Table S5.

Suppressor Screening

Suppressor screens were essentially performed as described above, except for the incorporation of a ~600 bp fragment that had been amplified by error-prone PCR using *Taq* DNA Polymerase.

Protein Purification and ATPase Activity Assay

Wild-type and Mini-Smc proteins were produced without tags in *E. coli* BL21-Gold(DE3) in Overnight Express Instant TB Medium (Merck Millipore) for 17 hr at 24°C. Cells were resuspended in lysis buffer (50 mM Tris-HCl pH 7.5, 150 mM NaCl, 1 mM EDTA, 1 mM DTT, 10% sucrose) and sonicated. The soluble phase was loaded on a HiTrap Blue HP 5 mL column (GE Healthcare) and was eluted with a linear gradient of buffer containing 1 M NaCl. The main peak elution fractions where diluted in buffer (50 mM Tris-HCl pH 7.5, 1 mM EDTA, 1 mM DTT) to a conductivity equivalent of 50 mM NaCl (≈8 mS/cm). The sample was loaded on a HiTrap Heparin HP 5 mL column (GE Healthcare) and was eluted with a linear gradient of buffer containing 2 M NaCl. The main peak fractions where pooled and concentrated to 2 mL in an Amicon Ultra-15 Centrifugal Filter Unit (Merck Millipore). The sample was loaded on a XK 16/70 Superose 6 PG column (GE Healthcare) in gel filtration buffer (50 mM Tris-HCl pH 7.5, 100 mM NaCl, 1 mM EDTA, 1 mM DTT). Main peak fractions where pooled, concentrated to 8 mg/mL and stored at −80°C. Protein concentration was determined by absorbance using theoretical molecular weight and molar absorptivity values.

The ATPase assay was carried out on a Synergy Neo Hybrid Multi-Mode Microplate reader (BioTek) monitoring the oxidation of NADH by absorbance at 340 nm in a pyruvate kinase/lactate dehydrogenase coupled reaction (Kornberg and Pricer, 1951). The final protein concentration in the assay was 0.3 μM in assay buffer (50 mM HEPES-KOH pH 7.5, 50 mM NaCl, 2 mM $MgCl_2$, 1 mM NADH, 1 mM ATP), and measurements were carried out at 25°C.

Site-specific in vivo Cross-linking

Cultures of 200 mL SMG were inoculated to OD_{600} = 0.004 and grown to OD_{600} = 0.02 at 37°C. Cells were harvested by filtration, washed in cold PBS + 0.1% glycerol (PBSG), and split into three aliquots of 0.85 OD units. Cells were re-suspended in 200 μL PBSG and cross-linked with 0.5 mM BMOE for 10 min on ice. The reaction was quenched by the addition of 14 mM 2-mercaptoethanol. Cells were pelleted and re-suspended in 30 μL of PBSG containing 75 U/mL ReadyLyse Lysozyme, 750 U/mL Sm DNase, 5 μM HaloTag TMR Substrate and protease inhibitor cocktail (Sigma). Lysis was performed at 37°C for 15 min. Then, 10 μL of 4X LDS-PAGE buffer were added, samples were incubated for 5 min at 95°C and resolved by SDS-PAGE. Gels were imaged on a Typhoon FLA9000 (GE Healthcare) with Cy3 DIGE filter setup.

Chromosome Entrapment Assay

The chromosome entrapment assay measures the co-purification of covalently circularized Smc–ScpAB with the chromosome (Wilhelm et al., 2015). Cells were grown, cross-linked and quenched as described above, except for the use of 3.75 OD units cell mass, 1 mM BMOE, 28 mM 2-mercaptoethanol and a reaction volume of 100 μL. ReadyLyse Lysozyme (400 U), protease inhibitor and HaloTag Oregon Green substrate (1 μM final) were added. The cell suspension was mixed immediately in a 1:1 ratio with a 2% solution of Low Melt Agarose (BioRad) equilibrated at 70°C and was cast into 100 μL agarose plugs using plug molds (BioRad). Agarose plugs were incubated for 20 min at 37°C protected from light, and then loaded into the wells of a 6% SDS-PAGE Tris-glycine gel. The gel was run for 60 min at 25 mA protected from light.

Agarose plugs were then re-extracted from the PAGE gel and transferred into 1.5 mL Eppendorf tubes. 1 mL of Wash Buffer ('WB': 0.01 mM EDTA, 0.5 mM Tris, 0.5 mM $MgCl_2$, 0.01% SDS) was added per agarose plug. Plugs were incubated for 10 min with gentle agitation protected from light. This step was repeated once. Wash buffer was then discarded and replaced by 100 μL fresh WB supplemented with 50 U of Sm DNase. Plugs were incubated at 37°C for 30 min. Plugs were melted at 85°C for 2 min under vigorous agitation. The samples were frozen at −80°C and stored overnight.

Samples were then thawed, centrifuged for 10 min at 4°C and 14,000 × g and transferred to a 0.45 μm CoStar Spin-X Tube Filter (Corning) and spun for 1 min at 10,000 × g. The flow-through was concentrated in a Speed Vac (Thermo Scientific, no heating, 2.5 hr running time). The concentrated sample was re-suspended in LDS Sample Buffer (NuPage) containing 200 mM DTT and heated for 3 min at 70°C. Samples were loaded on a 3%–8% Tris-Acetate gel (Life Technologies) and run for 2.5 hr at 35 mA per gel at 4°C. Gels were scanned on a Typhoon scanner (FLA 9000, GE Healthcare) with Cy2-DIGE filter setup.

Chromatin Immunoprecipitation

Cultures of 200 mL SMG were inoculated to OD_{600} = 0.004 and grown to OD_{600} = 0.02 at 37°C. Cells were fixed by addition of 20 mL of buffer F (50 mM Tris-HCl pH 7.4/24°C, 100 mM NaCl, 0.5 mM EGTA pH 8.0/24°C, 1 mM EDTA pH 8.0/24°C, 10% Formaldehyde) and incubation for 30 min at room temperature. Cells were harvested by filtration and washed in PBS. A cell mass corresponding to 2 OD units was re-suspended in 1 mL TSEMS (50 mM Tris pH 7.4/24°C, 50 mM NaCl, 10 mM EDTA pH 8.0/24°C, 0.5 M sucrose, protease inhibitor cocktail) containing 6 mg/mL lysozyme. Protoplasting was done by shaking at 37°C for 30 min. Protoplasts were washed once in 2 mL TSEMS, re-suspended in TSEMS, split into 3 aliquots and pelleted. Pellets were frozen in liquid nitrogen and stored at −80°C.

Pellets were re-suspended in 1 mL buffer L (50 mM HEPES-KOH pH 7.5/24°C, 140 mM NaCl, 1 mM EDTA pH 8.0/24°C, 1% Triton X-100, 0.1% Na-deoxycholate) containing 0.1 mg/mL RNase A and protease inhibitor cocktail. The suspension was sonicated in a Covaris E220 water bath sonicator for 5 min at 4°C, 100 W, 200 cycles, 10% load and filling level 0. The extract was centrifuged at 4°C and 20,000 × g and 100 μL were kept as input reference. For immunoprecipitation, 750 μL of the extract were loaded on 50 μL Dynabeads Protein-G charged with 50 μL Anti-ScpB antiserum and incubated for 2 hr on a wheel at 4°C. Beads were washed at room temperature in 1 mL each of buffer L, buffer L5 (buffer L containing 500 mM NaCl), buffer W (10 mM Tris-HCl pH 8.0/24°C, 250 LiCl, 0.5% NP-40, 0.5% Na-Deoxycholate, 1 mM EDTA pH 8.0/24°C) and buffer TE (10 mM Tris-HCl pH 8.0/24°C, 1 mM EDTA pH 8.0/24°C). Beads were resuspended in 520 μL buffer TES (50 mM Tris-HCl pH 8.0/24°C, 10 mM EDTA pH 8.0/24°C, 1% SDS). The reference sample was mixed with 100 μL buffer L, 300 μL buffer TES and 20 μL 10% SDS. Cross-links were reversed over-night at 65°C with shaking.

For phenol/chloroform extraction, samples were cooled to room temperature, vigorously mixed with 500 μL phenol equilibrated with buffer (10 mM Tris-HCl pH 8.0, 1 mM EDTA) and centrifuged for 10 min at 20,000 × g. Then, 450 μL of the supernatant was vigorously mixed with 450 μL chloroform and centrifuged for 10 min at 20,000 × g. For DNA precipitation, 400 μL of the supernatant were mixed with 1.2 μL GlycoBlue, 40 μL of 3 M Na-Acetate pH 5.2/24°C and 1 mL ethanol and incubated for 20 min at −20°C. Samples were centrifuged at 4°C and 20,000 × g for 10 min, and the precipitate was washed in 500 μL of 70% ethanol, dissolved in 250 μL buffer PB (QIAGEN) for 15 min at 55°C, purified with a PCR purification kit (QIAGEN), and eluted in 50 μL buffer EB.

For qPCR, samples were diluted in water (1:10 for IP and 1:100 for input), and duplicate 10 μL reactions (5 μL master mix, 1 μL of 3 μM primer mix, 4 μL sample) were run in a Rotor-Gene Q device (QIAGEN) using NoROX SYBR MasterMix (Takyon) and the primer pairs listed in Table S2.

For deep-sequencing, DNA was fragmented to ~200 bp and libraries were prepared using the Ovation Ultralow Library Systems V2 Kit (NuGEN) with 15 PCR cycles. Single-read sequencing was performed on a HiSeq 3000 (Illumina) with 150 bp read length.

Transposon Insertion Screen

A modified EZ-Tn5 transposon (Epibio) containing *Bsg*I restriction sites was randomly inserted into a double-crossover targeting construct for the endogenous *smc* locus (pSG1134). A fragment that reached to the stop codon but lacked the first ~8% of the coding sequence was cut from the primary library, purified from backbone and insert-free fragments by gel electrophoresis, and subcloned into the parental vector. Then, the transposon cassette was replaced in a *Bsg*I Golden Gate reaction by a short sequence permitting translation in either direction in all three reading-frames (CTGTCTGGACCGGGAGGCGGAGGAGGCAGACAG). The library was treated with *Xho*I to remove residual transposon containing plasmids and was amplified in *E. coli*. The library was transformed into a *smc* deletion strain and viable transformants were selected on ONA with antibiotics. Candidates were streaked for single colonies, the inserts were mapped by PCR and characterized by Sanger sequencing. Insert positions of viable isolates are listed in Table S6.

For deep-sequencing of the input library, a sequencing library was prepared using the NEXTflex PCR-Free Library Prep Kit (Bioo Scientific). Fragment size after fragmentation and sizing was ~400 bp. Single-read sequencing was performed on a HiSeq 3000 (Illumina) with 150 bp read length.

QUANTIFICATION AND STATISTICAL ANALYSIS

Analysis of Cross-Linking Efficiencies

Protein bands were quantified in Wolfram Mathematica. Background at the band was estimated with a moving median filter and subtracted. Credible intervals for cross-linking experiments were estimated from posterior distributions using a normally distributed likelihood with mean μ and standard deviation σ, a uniform prior over [0, 1] for μ and a $1/\sigma$^2 prior for σ. All data points for technical replicates are shown in the figures. The definition of the center and precision measurements are reported in the figure legends (mean, standard deviation, 95% credible interval).

Fourier Analysis

For Fourier analysis, the growth datasets of truncation and extension screens were normalized to their 95% quantiles and merged by averaging at overlapping positions. Then, the region between coiled-coil lengths of 253-435 AA was used to compute the discrete Fourier transform.

Steady-State Enzyme Kinetics

Time series were corrected for data from a protein-free reference. Absorbance differences were converted to concentration differences using the molar absorptivity of NADH. The specific steady-state reaction rate *v* was determined from the slope of a linear fit to the time series divided by the protein concentration. Substrate-concentration dependent reaction rates *v* were fit to the Hill model:

$$v(c) = \frac{c^n \, v_{max}}{c^n + K_{0.5}^n}$$

where *c* is the ATP concentration, *n* is the degree of cooperativity between ATP binding sites, v_{max} is the maximum rate, and $K_{0.5}$ is the ATP concentration at half-maximum rate. Parameter and precision estimates (mean and standard deviation) were computed from best-fit parameters to multiple independent titration series (Table S3).

Analysis of qPCR Data

qPCR data were fit to a 5-parameter logistic model (Spiess et al., 2008):

$$f(t) = (c - b)(Exp(a(t - d)) \; + 1)^{-e} + b$$

where *t* is the time in cycles and *a*, *b*, *c*, *d*, *e* are model parameters. The threshold cycle (C_T) was defined as the position of the second-derivative maximum of the fit:

$$C_T = -\frac{\log\left(\frac{1}{2}\left(-\sqrt{5e^2 + 6e + 1} + 3e + 1\right)\right) - ad}{a}$$

Amplification efficiencies were not determined and IP/input ratios were calculated as $\alpha\, 2^{\Delta CT}$, where $\Delta C_T = C_T$ (Input) – C_T (IP) and α is a constant determined by extraction volumes and sample dilutions. Data are presented as the mean of duplicate PCR reactions.

Analysis of ChIP-seq Data

Deep-sequencing data for the immunoprecipitate were mapped to the *B. subtilis* reference genome (centered on its first coordinate) using Bowtie 2 (Langmead and Salzberg, 2012). Reads were filtered for mapping quality (MAPQ) greater than 10, reduced to bins of 100 bp, smoothed with an averaging sliding window of 3 bins, and normalized for total read count. A 400 bp region centered at genome coordinate 3776100 was excluded from analysis due to an apparent amplification artifact in the negative control sample. Data are presented in reads per million (rpm).

For ratiometric analysis, the reduced data of each sample was compared to the reduced data of the wild-type sample (Minnen et al., 2016). For each bin, the larger value was divided by the smaller, and the resulting ratio was plotted above the coordinate axis for value(mutant) $\geq$ value(WT) and below the axis otherwise. Ratios above 20 were treated as outliers and set to 1.

Analysis of Insertion Library Sequencing Data

Reads containing the insert were identified and mapped to the parental vector pSG1134 with Wolfram Mathematica. We observed 78,369 reads containing the insert. Of those, 87.3% mapped to the targeted region. In this region, 27.6% of codons were hit at least once (Figure S6A) (virtually 100% are expected at this sequencing depth for a uniform distribution of insertion events). We detected a transposition bias in one of the two possible orientations, but no bias with respect to the reading frame (Figure S6A). The number of the first codon directly at or upstream of the insertion site not leading to an amino acid substitution was defined as the insertion site at protein level.

Permutation Test

To test for a difference in enrichment of coiled-coil inserts, library and isolate samples of the transposon screen were subjected to an approximate permutation test. First, for each sample we computed the fraction f of inserts mapping to the coiled coil, and used $r = f_{isolates}/f_{library} = 0.6016$ as a test statistic. We then pooled the samples, resampled 1000 times without replacement, and computed r for each resampling. We did not observe a single event where $r \leq 0.6016$. We estimate that $p < 0.001$ and infer that the viable isolates are depleted from inserts in their coiled-coil arm relative to inserts in other regions of the protein.

Kernel Density Estimation

Modes of coiled-coil length distributions were obtained from kernel density estimates using a Gaussian kernel with a bandwidth of 8 AA (4 AA for display).

DATA AND SOFTWARE AVAILABILITY

ChIP-seq data reported in this paper has been deposited at the NCBI Sequence Read Archive under the accession number SRA: SRP094054.

Deep-sequencing data of the insertion library reported in this paper has been deposited at the NCBI Sequence Read Archive under the accession number SRA: SRP094088.

A Wolfram Language package for the analysis of insertion screens is available at https://github.com/fbuermann/InsertionMapping.

Molecular Cell, Volume *65*

Supplemental Information

Tuned SMC Arms Drive Chromosomal Loading of Prokaryotic Condensin

Frank Bürmann, Alrun Basfeld, Roberto Vazquez Nunez, Marie-Laure Diebold-Durand, Larissa Wilhelm, and Stephan Gruber

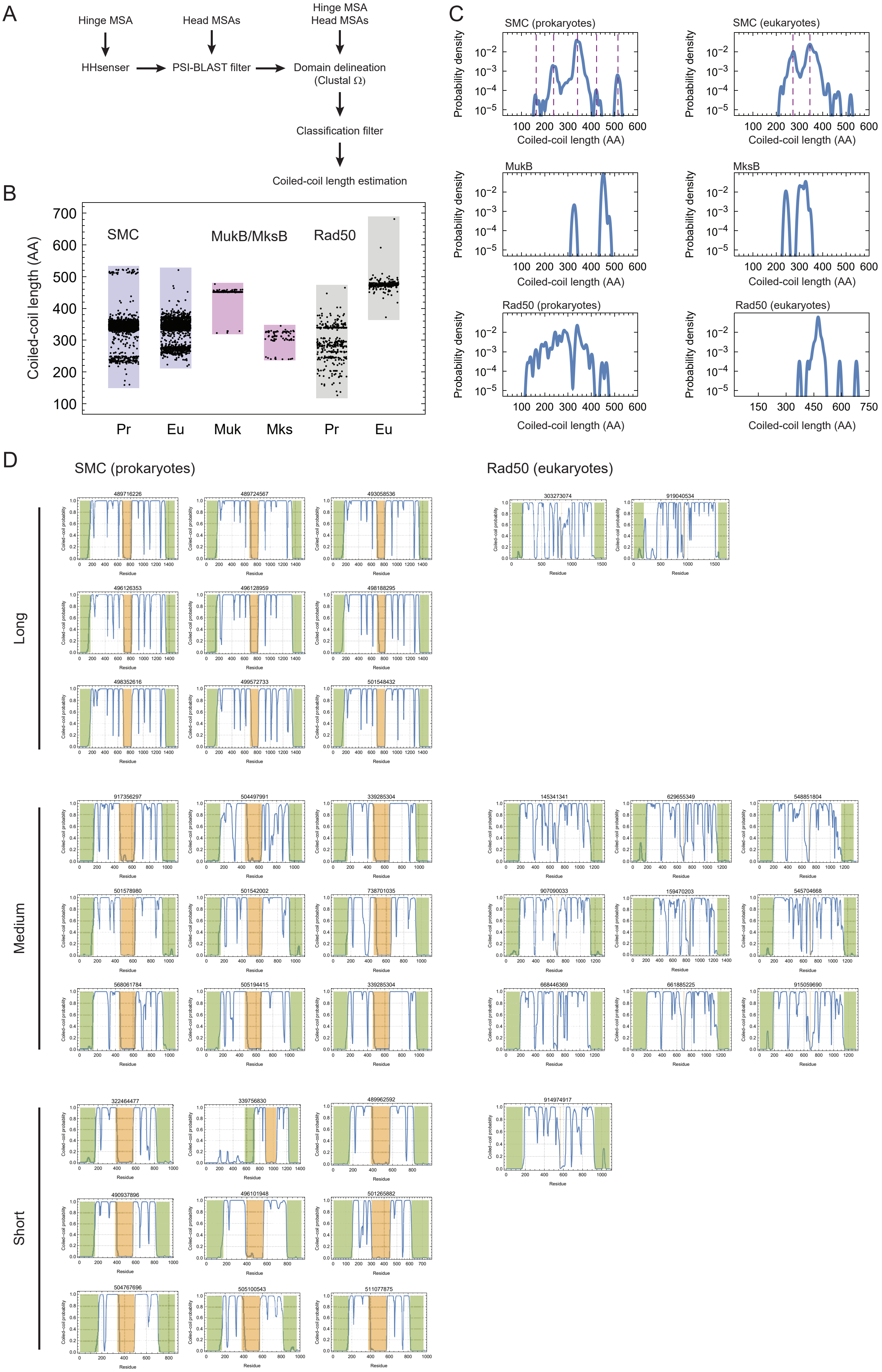
A
Hinge MSA
Head MSAs
Hinge MSA
Head MSAs
HHsenser
PSI-BLAST filter
Domain delineation
(Clustal Ω)
Classification filter
Coiled-coil length estimation
B
Coiled-coil length (AA)
SMC
MukB/MksB
Rad50
Pr
Eu
Muk
Mks
Pr
Eu
C
SMC (prokaryotes)
SMC (eukaryotes)
MukB
MksB
Rad50 (prokaryotes)
Rad50 (eukaryotes)
Probability density
Coiled-coil length (AA)
D
SMC (prokaryotes)
Rad50 (eukaryotes)
Long
Medium
Short
Coiled-coil probability
Residue

Figure S1

Figure S1. Coiled-coil length distributions of SMC and SMC-like proteins. Related to Figure 1.

(A) Flow scheme for the estimation of coiled-coil length distributions.

(B) Arm length distributions for different groups of SMC and SMC-like proteins. Also shown in Figure 1B: SMC prokaryotes, n = 3337; SMC eukaryotes, n = 1659. Additional data: MukB, n = 179; MksB, n = 75; Rad50 prokaryotes, n = 365; Rad50 eukaryotes, n = 306.

(C) Kernel density estimates for data shown in B. Top panels also shown in Figure 1C.

(D) Example profiles for selected SMC family sequences with short, medium and long coiled-coil arms. Marcoil coiled-coil predictions are shown in blue colors, and regions identified by the sequence alignment approach described in A are indicated in green (HeadN and HeadC) and orange (Hinge), respectively. GenBank identifiers are indicated on top of the plots.

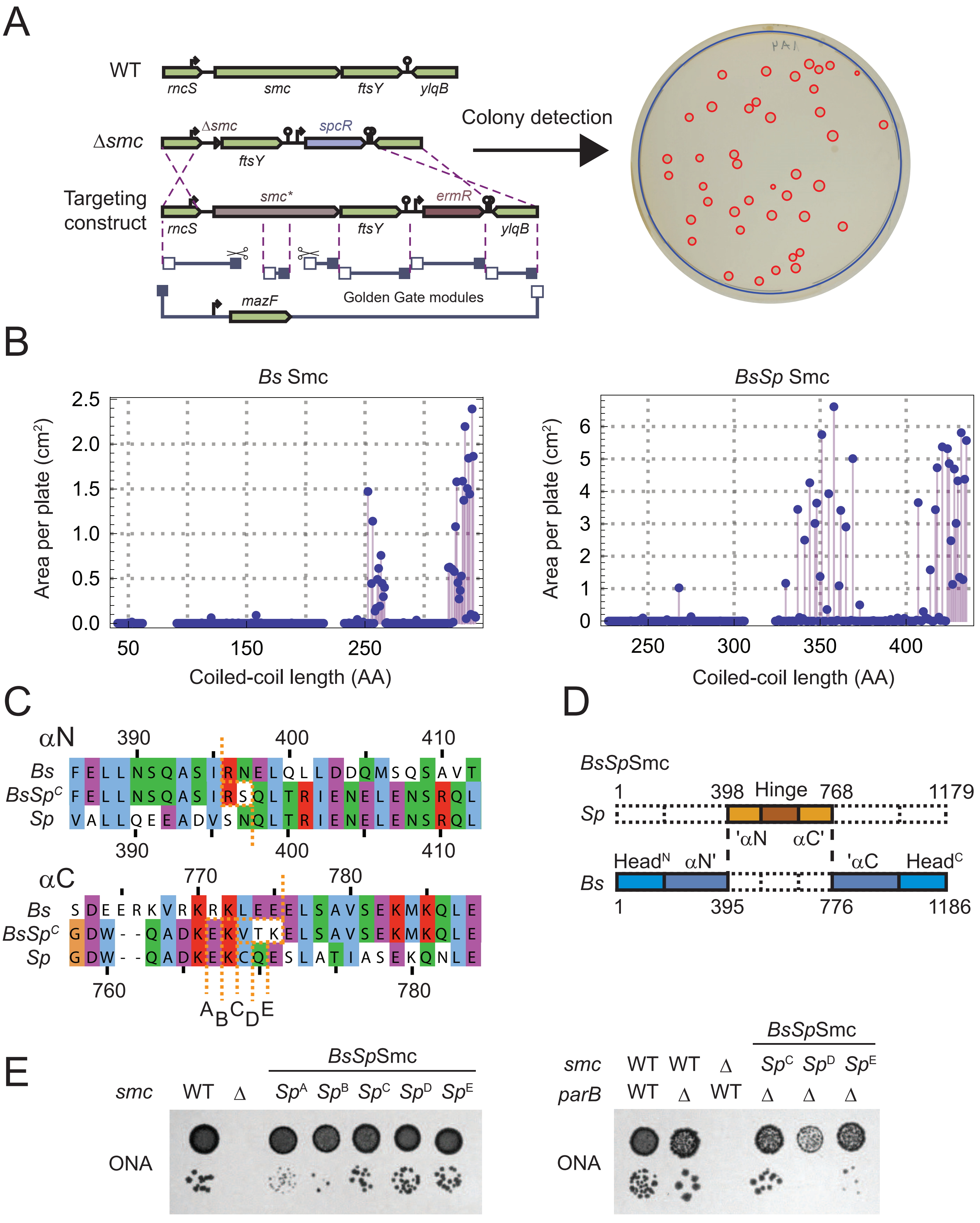
A
WT
rncS
smc
ftsY
ylqB
Δsmc
Δsmc
spcR
ftsY
Targeting construct
smc*
ermR
rncS
ftsY
ylqB
mazF
Golden Gate modules
Colony detection
B
Bs Smc
BsSp Smc
Area per plate (cm²)
Coiled-coil length (AA)
C
αN
αC
Bs
BsSp^C
Sp
A B C D E
D
BsSpSmc
Sp
Bs
Hinge
'αN
αC'
Head^N
αN'
'αC
Head^C
1
398
768
1179
395
776
1186
E
smc
parB
WT
Δ
BsSpSmc
Sp^A
Sp^B
Sp^C
Sp^D
Sp^E
ONA

Figure S2

Figure S2. Coiled-coil truncation and extension screens. Related to Figure 2.

(A) Gene targeting strategy.

(B) Bacterial growth per plate obtained for the coiled-coil shortening screen (left panel) and the coiled coil extension screen (right panel) shown in Figure 2C.

(C) Sequence alignment for the seam of different *BsSp* chimeras. *Bs* Smc and *Sp* Smc were combined with slightly altered connections in the C-terminal coiled-coil strand (bottom), whereas the N-terminal strand was kept constant (top). Connections of the variants are indicated by dotted lines.

(D) Scheme for construction of the *BsSp* Smc hybrid protein used in Figure 2C.

(E) Spot dilutions for *BsSp* Smc variants in the presence (left) or absence (right) of the *parB* gene. Deletion of *parB* sensitizes strains with hypomorphic *smc* alleles (Gruber and Errington, 2009). Variant C was used for the screen shown in Figure 2C.

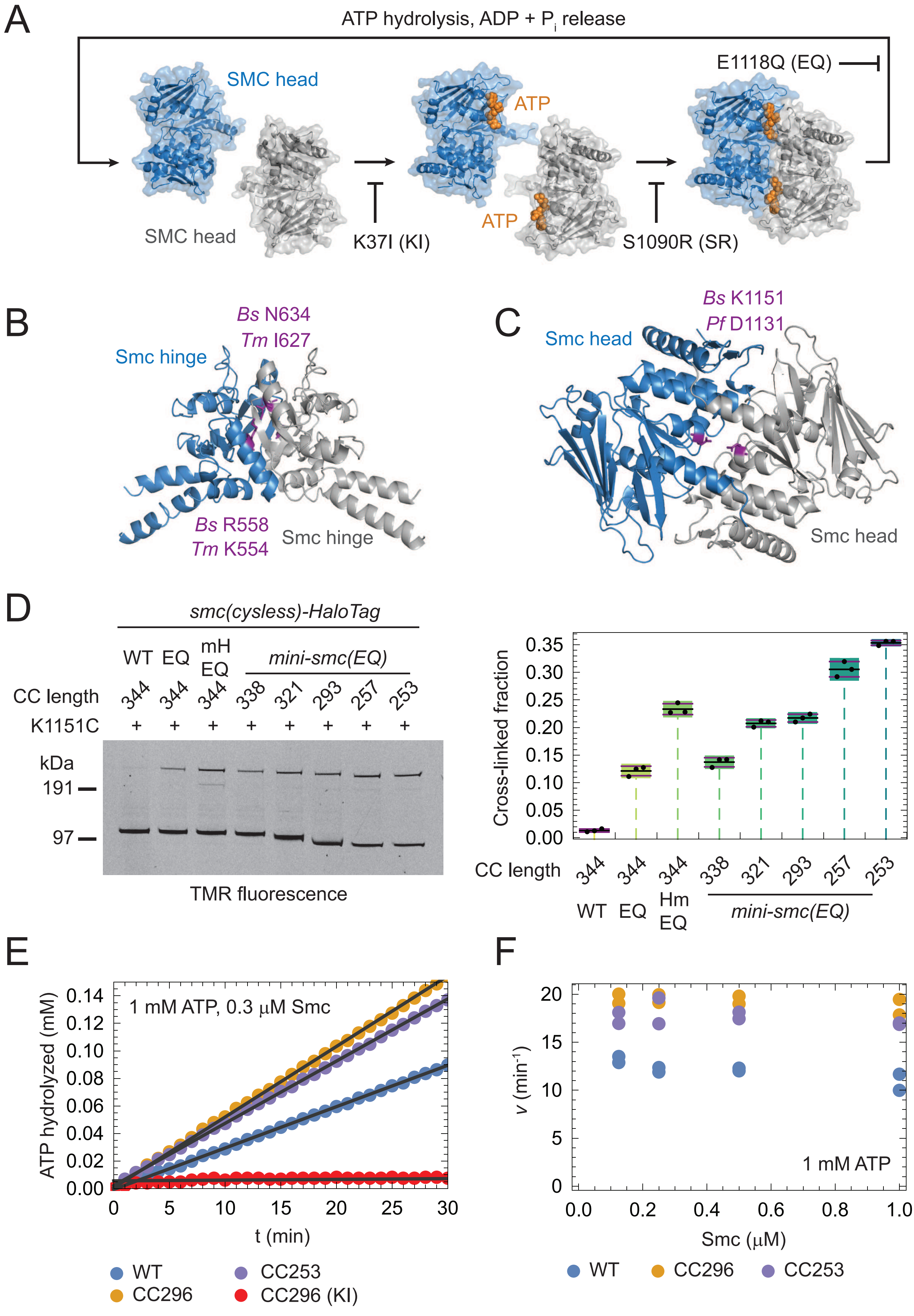

A
ATP hydrolysis, ADP + P i release
SMC head
ATP
E1118Q (EQ)
SMC head
K37I (KI)
ATP
S1090R (SR)
B
Bs N634
Tm I627
Smc hinge
Bs R558
Tm K554
Smc hinge
C
Bs K1151
Pf D1131
Smc head
Smc head
D
smc(cysless)-HaloTag
WT EQ mH EQ
mini-smc(EQ)
CC length 344 344 344 338 321 293 257 253
K1151C + + + + + + + +
kDa
191
97
TMR fluorescence
Cross-linked fraction
0.35 0.30 0.25 0.20 0.15 0.10 0.05 0.00
CC length 344 344 344 338 321 293 257 253
WT EQ Hm EQ
mini-smc(EQ)
E
1 mM ATP, 0.3 μM Smc
ATP hydrolyzed (mM)
0.14 0.12 0.10 0.08 0.06 0.04 0.02 0.00
0 5 10 15 20 25 30
t (min)
WT
CC296
CC253
CC296 (KI)
F
v (min-1)
20 15 10 5 0
1 mM ATP
0.0 0.2 0.4 0.6 0.8 1.0
Smc (μM)
WT
CC296
CC253

Figure S3

Figure S3. Site-specific cross-linking of interfaces in Mini-Smc variants. Related to Figure 3.

(A) Overview of the SMC ATPase cycle. *Bs* Smc mutations interfering with the indicated steps are: K37I (KI), blocks ATP binding; S1090R (SR), blocks head engagement; E1118Q (EQ), blocks ATP hydrolysis. Head structures are from (Lammens et al., 2004).

(B) The location of the reporter residues R558C/N634C (Bürmann et al., 2013) is mapped onto the *Tm* hinge crystal structure (PDB: 1GXL, top).

(C) The head engagement reporter residue K1151C (Minnen et al., 2016) is mapped onto the *P. furiosus* (*Pf*) head crystal structure (PDB: 1XEQ).

(D) *In vivo* site-specific cross-linking of Mini-Smc variants at the head interface. EQ, E1118Q allows ATP binding but impairs hydrolysis; mH, monomeric hinge (Hirano and Hirano, 2002; Minnen et al., 2016). As in Figure 3B.

(E) Examples of ATPase time course measurements. Representative data for selected Smc variants at 1 mM ATP are shown.

(F) Specific activity of selected Smc variants at 1 mM ATP and variable protein concentration.

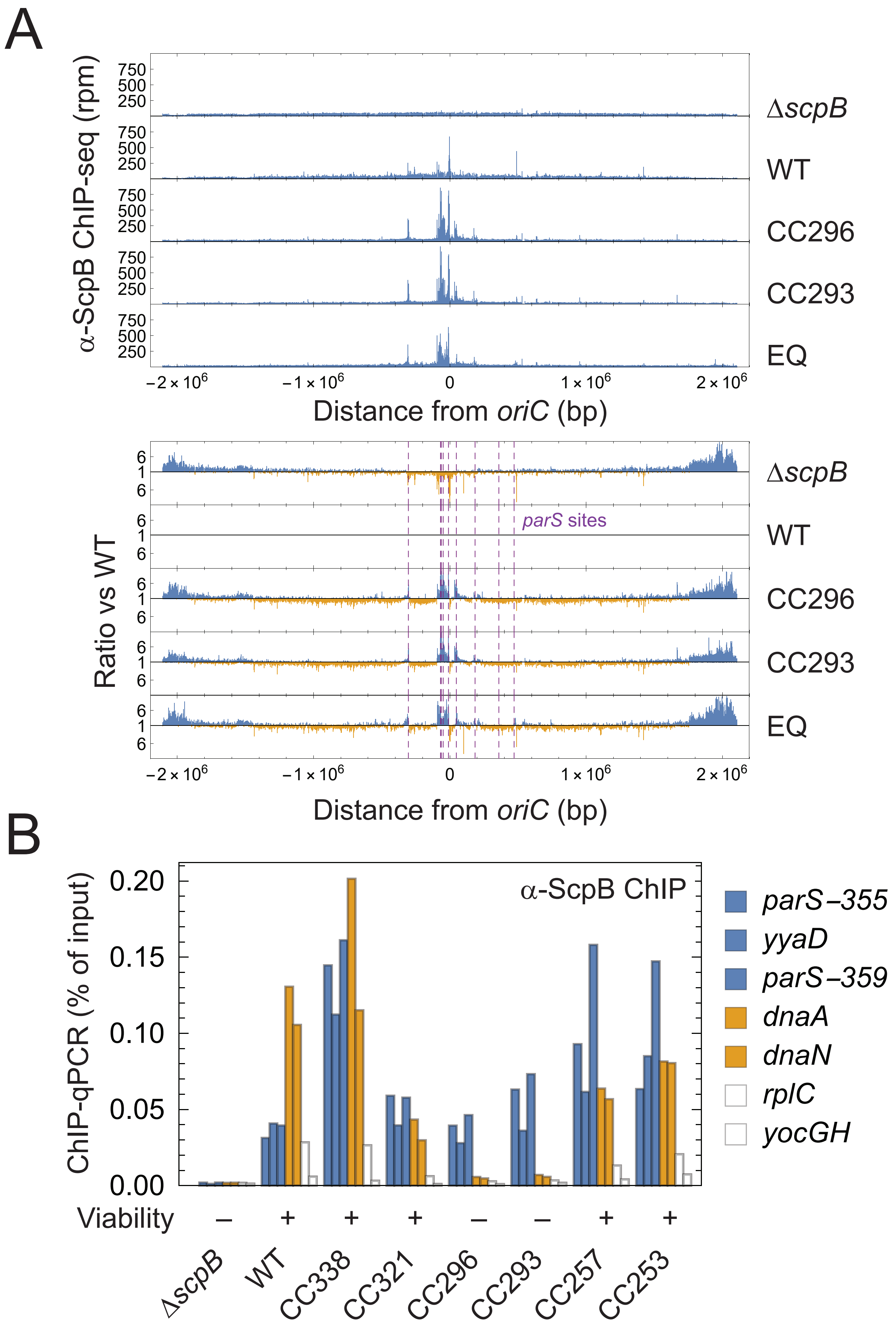
A
α-ScpB ChIP-seq (rpm)
750
500
250
ΔscpB
WT
CC296
CC293
EQ
−2 × 10^6
−1 × 10^6
0
1 × 10^6
2 × 10^6
Distance from oriC (bp)
Ratio vs WT
6
1
6
parS sites
B
ChIP-qPCR (% of input)
0.20
0.15
0.10
0.05
0.00
α-ScpB ChIP
parS−355
yyaD
parS−359
dnaA
dnaN
rplC
yocGH
Viability
–
+
+
+
–
–
+
+
ΔscpB
WT
CC338
CC321
CC296
CC293
CC257
CC253

Figure S4

Figure S4. Chromosomal localization of Mini-Smc variants. Related to Figure 4.

(A) Chromosome-wide ChIP-seq profiles for different Smc variants. Sequence distributions for the immunoprecipitate (top) and ratiometric analysis against the wild-type profile (bottom) are shown. Locations of *parS* sites are indicated by purple dashed lines.

(B) ChIP-qPCR against ScpB for *mini-smc* strains. Loci close to Smc loading sites are colored in orange, loci close to the replication origin are blue and chromosomal arm positions are white (see also Figure 4B).

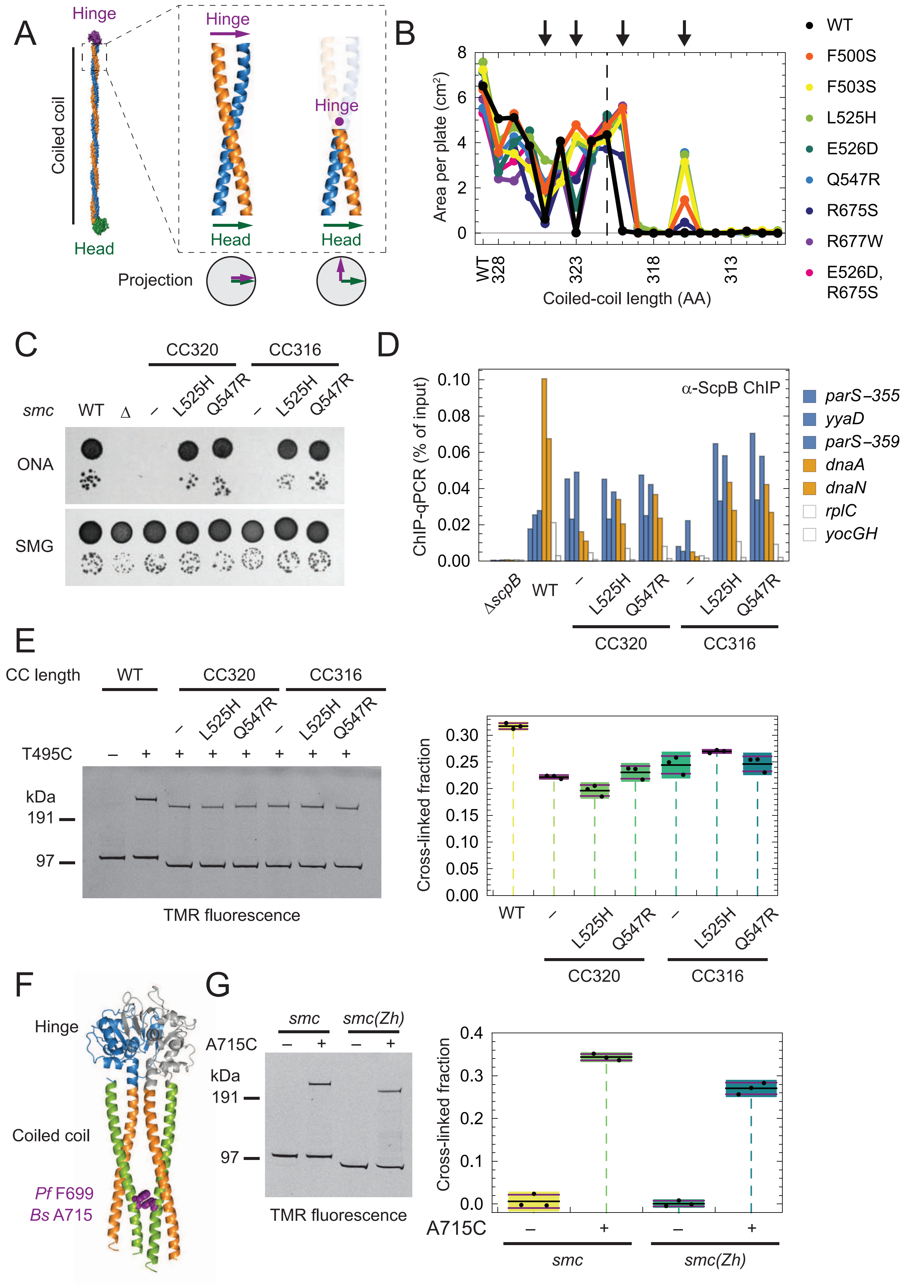

A
Hinge
Coiled coil
Head
Hinge
Head
Hinge
Head
Projection
B
WT
F500S
F503S
L525H
E526D
Q547R
R675S
R677W
E526D, R675S
Area per plate (cm²)
Coiled-coil length (AA)
WT 328
323
318
313
C
CC320
CC316
smc
WT
Δ
L525H
Q547R
ONA
SMG
D
α-ScpB ChIP
ChIP-qPCR (% of input)
parS-355
yyaD
parS-359
dnaA
dnaN
rplC
yocGH
ΔscpB
CC320
CC316
E
CC length
T495C
kDa
191
97
TMR fluorescence
Cross-linked fraction
F
Hinge
Coiled coil
Pf F699
Bs A715
G
smc
smc(Zh)
A715C
kDa
191
97
TMR fluorescence
Cross-linked fraction

Figure S5

Figure S5. Characterization of Mini-Smc suppressors and the Smc(Zh) chimera. Related to Figure 5.

(A) Modifications in Smc arm length might alter hinge/head orientation by changing the phase-relationship between coiled-coil ends.

(B) Confirmation of suppressor mutations. The indicated mutations were introduced into different *mini-smc* variants by an approach similar to the one shown in Figure 2A, and total colony area per plate of transformants was assessed.

(C) Spot dilutions of selected *mini-smc* suppressor strains. As in Figure 2E.

(D) ChIP-qPCR against ScpB for *mini-smc* strains containing suppressor mutations. Loci close to Smc loading sites are colored in orange, loci close to the replication origin are blue and chromosomal arm positions are white (see also Figure 4B).

(E) Site-specific *in vivo* cross-linking at the hinge-proximal coiled-coil residue T495C in suppressed Mini-Smc variants (Soh et al., 2015). As in Figure 3A.

(F) Rod conformation of the hinge-proximal coiled coil (*Pf* Smc, PDB: 4RSJ). The reporter residue A715C is indicated in purple (Soh et al., 2015). The region that has been modified in the truncation screen is shown in orange and green, the constant hinge region is shown in blue and gray. For experiments involving the Rad50 Zinc hook, the constant hinge region was replaced by the hook.

(G) Association of the hinge-proximal coiled coil in the Smc(Zh) chimera as determined by site-specific *in vivo* cross-linking. See Figure S5F for location of the reporter residue. As in Figure 3A.

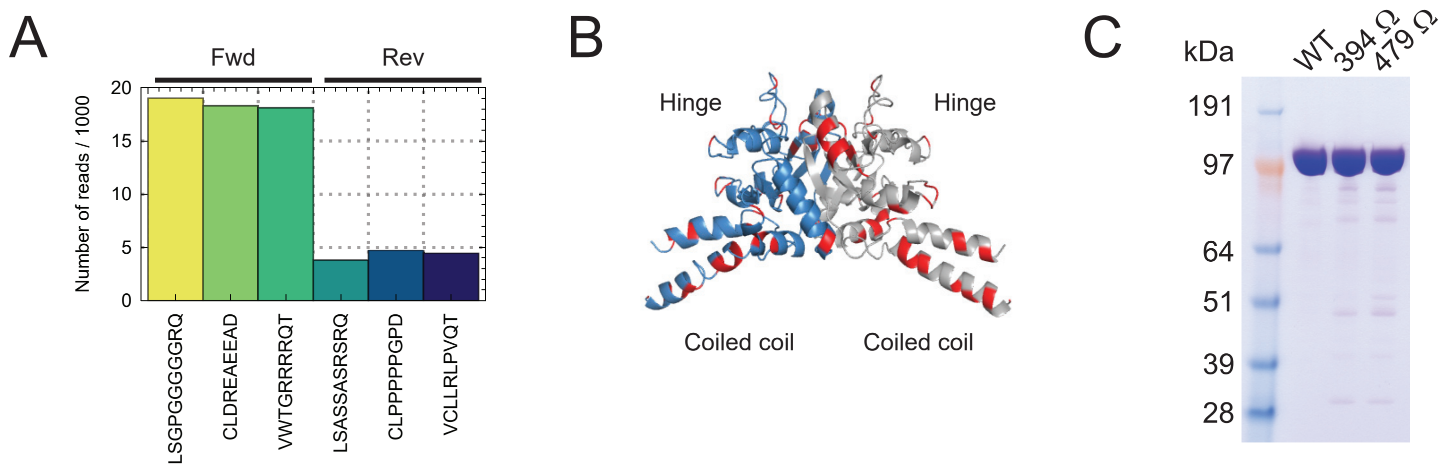
A
Fwd
Rev
Number of reads / 1000
20
15
10
5
0
LSGPGGGGRQ
CLDREAEEAD
VWTGRRRQT
LSASSASRSRQ
CLPPPPPGPD
VCLLRLPVQT
B
Hinge
Hinge
Coiled coil
Coiled coil
C
kDa
WT
394 Ω
479 Ω
191
97
64
51
39
28

Figure S6

Figure S6. Transposon insertion screen. Related to Figure 6.

(A) Read counts for all six different frames of the inserted sequence.
(B) Positions of peptide insertions resulting in functional proteins are mapped onto the *Tm* hinge structure (PDB: 1GXL).
(C) Purification of Smc variants with peptide insertions in the coiled-coil arm. Purified fractions were analysed by SDS-PAGE and Coomassie staining.

Supplemental tables

Table S1, related to Figures 1- 6. Strain usage.

Figure	Strains
2E	BSG1002, BSG1007, BSG2088, BSG2089, BSG2090, BSG2091, BSG2092, BSG2093, BSG2094, BSG2104
2F	BSG1002, BSG1007, BSG2348, BSG2349, BSG2355, BSG2350, BSG2351, BSG2352, BSG2353, BSG2356, BSG2354
3A	BSG1360, BSG1638, BSG2118, BSG2119, BSG2403, BSG2120, BSG2121, BSG2122
3B	BSG1457, BSG1488, BSG1600, BSG2511, BSG2531, BSG2408, BSG2135
4A	BSG1891, BSG1002, BSG2090, BSG2091, BSG1008
4B	BSG1891, BSG1002, BSG1008, BSG1046, BSG2090, BSG2409, BSG2091, BSG2410
4C	BSG1782, BSG1784, BSG1786, BSG2617, BSG2618, BSG2619, BSG2620
5B	BSG1002, BSG2479, BSG2578, BSG2579, BSG2580
5E	BSG1002, BSG1007, BSG1075, BSG2414, BSG2415, BSG2416, BSG2417, BSG2418, BSG2419
6B	BSG1002, BSG1007, BSG2017, BSG2018, BSG2021, BSG2026, BSG1835
6C	BSG1891, BSG1002, BSG2017, BSG2018, BSG2021, BSG2026, BSG1835
S3D	BSG1457, BSG1488, BSG1598, BSG2133, BSG2134, BSG2135, BSG2136, BSG2137
S4A	BSG1891, BSG1002, BSG2090, BSG2091, BSG1008
S4B	BSG1891, BSG1002, BSG2088, BSG2089, BSG2090, BSG2091, BSG2092, BSG2093
S5C	BSG1002, BSG1007, BSG2479, BSG2480, BSG2481, BSG2482, BSG2483, BSG2484
S5D	BSG1891, BSG1002, BSG2479, BSG2480, BSG2481, BSG2482, BSG2483, BSG2484
S5E	BSG1457, BSG2485, BSG2486, BSG2487, BSG2488, BSG2492, BSG2493, BSG2494
S5G	BSG1360, BSG1921, BSG2512, BSG2513

Table S2, related to STAR Methods. Primers used for qPCR.

Locus	Primer1	Primer2
parS-355	taattcatcatcgcgctcaa	aatgccgattacgagtttgc
yyaD	cttgcgatttttgcttctcc	acatcaccatacgtggacga
parS-359	aaaaagtgattgcggagcag	agaaccgcatctttcacagg
dnaA	gatcaatcggggaaagtgtg	gtagggcctgtggatttgtg
dnaN	gaattccttcaggccattga	gatttctggcgaattggaag
rplC	ttgacgacaagcgtgaaaag	ttcatacgcatccatttcca
yocGH	tccatatcctcgctcctacg	attctgctgatgtgcaatgg

Table S3, related to Figure 3. Enzymological parameters for Smc constructs with short coiled-coils or arm insertions.

Values are given as mean ± s.d. of best-fit values obtained from the indicated number of replicate experiments.

Construct	v_{max}	$K_{0.5}$	n	Replicates	Plasmid
WT	16.2 ± 2.1	0.48 ± 0.13	1.047 ± 0.092	6	pSG1497
CC296	17.21 ± 0.72	0.0537 ± 0.0060	1.608 ± 0.082	4	pSG2914
CC293	16.6 ± 3.4	0.0460 ± 0.0075	1.74 ± 0.10	4	pSG2915
CC257	19.3 ± 1.3	0.137 ± 0.01	1.345 ± 0.059	4	pSG2916
CC253	18.5 ± 1.4	0.274 ± 0.038	1.145 ± 0.059	4	pSG2917
CC296 (K37I)	n.d.	n.d.	n.d.	4	pSG2920
CC293 (K37I)	n.d.	n.d.	n.d.	4	pSG2921
394Ω	21.22 ± 0.70	0.1189 ± 0.0023	1.347 ± 0.089	2	pSG2965
479Ω	17.86 ± 0.64	0.1001 ± 0.0025	1.4394 ± 0.0090	2	pSG2966

Tables S4, S5 and S6 are available as separate files.

Table S4, related to Figure 1. Prediction of coiled coil length in natural SMC sequences.

Table S5, related to Figure 2. Construction and viability of shortened and elongated Smc variants based on Smc, *BsSp* Smc and Smc(Zh).

Table S6, related to Figure 6. Transposon insertion screen: Functional Smc proteins with peptide insertions.
